# Transforming Growth Factor β Receptor Type 1 Is Essential for Female Reproductive Tract Integrity and Function

**DOI:** 10.1371/journal.pgen.1002320

**Published:** 2011-10-20

**Authors:** Qinglei Li, Julio E. Agno, Mark A. Edson, Ankur K. Nagaraja, Takashi Nagashima, Martin M. Matzuk

**Affiliations:** 1 Department of Pathology and Immunology, Baylor College of Medicine, Houston, Texas, United States of America; 2 Department of Molecular and Cellular Biology, Baylor College of Medicine, Houston, Texas, United States of America; 3 Department of Molecular and Human Genetics, Baylor College of Medicine, Houston, Texas, United States of America; 4 Department of Pharmacology, Baylor College of Medicine, Houston, Texas, United States of America; Stanford University School of Medicine, United States of America

## Abstract

The transforming growth factor β (TGFβ) superfamily proteins are principle regulators of numerous biological functions. Although recent studies have gained tremendous insights into this growth factor family in female reproduction, the functions of the receptors *in vivo* remain poorly defined. TGFβ type 1 receptor (TGFBR1), also known as activin receptor-like kinase 5, is the major type 1 receptor for TGFβ ligands. *Tgfbr1* null mice die embryonically, precluding functional characterization of TGFBR1 postnatally. To study TGFBR1–mediated signaling in female reproduction, we generated a mouse model with conditional knockout (cKO) of *Tgfbr1* in the female reproductive tract using anti-Müllerian hormone receptor type 2 promoter-driven Cre recombinase. We found that *Tgfbr1* cKO females are sterile. However, unlike its role in growth differentiation factor 9 (GDF9) signaling *in vitro*, TGFBR1 seems to be dispensable for GDF9 signaling *in vivo*. Strikingly, we discovered that the *Tgfbr1* cKO females develop oviductal diverticula, which impair embryo development and transit of embryos to the uterus. Molecular analysis further demonstrated the dysregulation of several cell differentiation and migration genes (e.g., *Krt12*, *Ace2*, and *MyoR*) that are potentially associated with female reproductive tract development. Moreover, defective smooth muscle development was also revealed in the uteri of the *Tgfbr1* cKO mice. Thus, TGFBR1 is required for female reproductive tract integrity and function, and disruption of TGFBR1–mediated signaling leads to catastrophic structural and functional consequences in the oviduct and uterus.

## Author Summary

Approximately 20% of infertile couples in the United States have unexplained causes. Many vital aspects of female fertility are regulated by a family of growth factors called the transforming growth factor β (TGFβ) superfamily. These factors exert their functions via specific receptors and downstream signal mediators. Perturbation of components in this pathway can lead to reproductive dysfunction. We identified a novel role for a TGFβ receptor (called TGFBR1) in female fertility. We demonstrated that female mice with disruption of *Tgfbr1* in the reproductive tract are unable to successfully conceive, although they can ovulate and produce fertilizable oocytes. Most importantly, these mice have a striking deformity in the oviduct, marked by the formation of oviductal outpouchings (diverticula) that prevent embryos from reaching the uterus. Concomitant aberrations in the uterine smooth muscle layers are additional features of mice lacking TGFBR1. Therefore, TGFBR1 is critical for the structural integrity and function of the female reproductive tract. Our model can be further exploited to study the development of smooth muscle cells of the female reproductive tract. Genetic mutations in *TGFBR1* or other TGFβ signaling machinery may lead to fertility defects in women.

## Introduction

The transforming growth factor β (TGFβ) superfamily, the largest family of secreted growth factors in mammals, is a conserved family of proteins that play key roles in diverse physiological and pathological processes [1]–[6]. The pathway consists of ligands, receptors, and SMAD transducers, and is tightly controlled by various regulatory layers such as ligand traps (e.g., noggin, follistatin, and gremlin), inhibitory SMADs (i.e., SMAD6 and SMAD7), as well as multiple interactive pathways that cross talk with TGFβ signaling proteins in a context-specific manner [7]–[9]. TGFβ ligands bind to their type 2 and type 1 receptors and activate intracellular SMAD proteins including receptor-regulated SMADs and common SMAD (SMAD4) to initiate signal transduction. Although more than 40 TGFβ family members have been discovered to date, there are only seven type 1 receptors (ACVRL1, ACVR1, BMPR1A, ACVR1B, TGFBR1, BMPR1B, ACVR1C) and five type 2 receptors (TGFBR2, AMHR2, ACVR2, ACVR2B, and BMPR2) in mammals [10], [11]. The receptor-regulated SMADs can be divided into TGFβ/activin responsive SMADs (i.e., SMAD2/3) and bone morphogenetic protein (BMP) responsive SMADs (i.e., SMAD1/5/8) based on the ligands with which they are associated in the signal transduction cascades [2], [7].

Recent studies have revealed that the TGFβ signaling pathway is critically involved in multiple reproductive events including, but not limited to, ovarian folliculogenesis [3], [12]–[15], cumulus cell expansion and ovulation [16]–[18], uterine decidualization [19], and embryo implantation [20]. Disturbances in TGFβ signaling have been shown to lead to severe pathological conditions such as cancer [1], [2], [4]–[6], [21]–[26], making it an appealing candidate pathway for therapeutic interventions. Early studies in our laboratory demonstrated that inhibin α is a tumor suppressor specific to the gonad and adrenal glands [21], highlighting the functional importance of TGFβ family proteins. Subsequent studies demonstrated that the BMP signaling pathway serves as a brake for ovarian tumor development [23], [26]. During recent years, significant progress has been made toward understanding the roles of this growth factor family in female reproduction [2], [3], [27]–[30]; however, the functions of the receptors *in vivo* remain poorly defined, partially due to receptor redundancy [26], [31]–[34] or lethal phenotypes of genetically engineered ubiquitous null mouse models.

TGFBR1 is the type 1 receptor for TGFβ ligands [2]. *In vitro*, TGFBR1 can also mediate the signaling of growth differentiation factor 9 (GDF9) [35], an oocyte-secreted protein required for early ovarian folliculogenesis, cumulus cell functions [12], [36]–[41], and oocyte developmental competence [42], [43]. The above evidence points to a possible role of TGFBR1 in female reproduction *in vivo*. However, the functional significance of TGFBR1 in the female reproductive tract is unknown because *Tgfbr1* null mice die embryonically [44].

Advances in gene targeting technology make it possible to dissect gene functions in specific tissues using a conditional gene inactivation (*Cre-loxP*) strategy [45]. Our laboratory has successfully utilized this technique to expand the understanding of reproductive functions of TGFβ signaling components [18], [23], [26], [46], [47]. In the current study, we generated a conditional knockout (cKO) of *Tgfbr1* in the female reproductive tract using anti-Müllerian hormone receptor type 2 (*Amhr2*)-Cre. We found that *Tgfbr1* cKO mice are sterile. Interestingly, instead of manifesting an overt ovarian phenotype, these mice develop striking oviductal and uterine phenotypes, thereby uncovering a novel role of TGFBR1–mediated signaling in female reproductive tract development and function.

## Results

### Generation of *Tgfbr1* Conditional Knockout Mice

**Figure 1:**
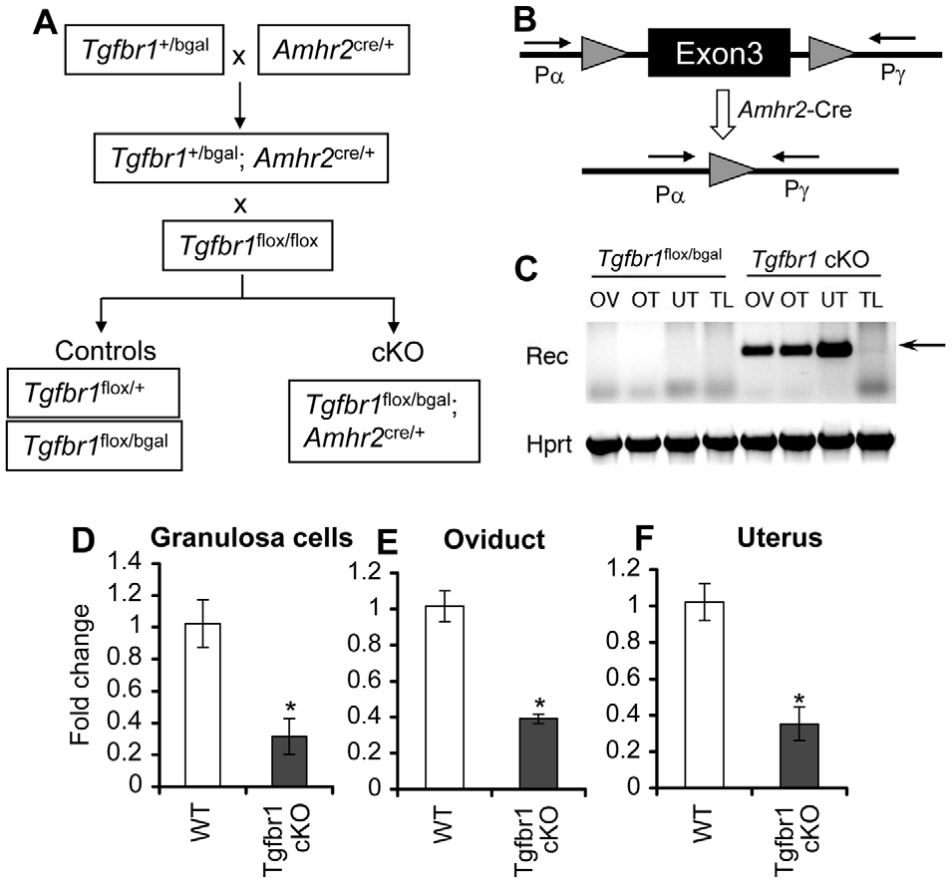
Generation of *Tgfbr1* conditional knockout mice. (A) Schematic representation of the breeding strategy for generating *Tgfbr1* cKO and control mice. To ensure maximal deletion of *Tgfbr1* and to visualize the localization of *Tgfbr1* in the female reproductive tract, the *Tgfbr1* ^bgal^ allele was used in the breeding. (B) Illustration of the *Tgfbr1* conditional allele with exon 3 flanked by two *loxP* sites. Primers Pα and Pγ detect the recombined allele of *Tgfbr1*. (C) Recombination of *Tgfbr1* floxed alleles in the genomic DNA of *Amhr2*-Cre target tissues. The recombined bands were detectable in the ovary (OV), oviduct (OT), and uterus (UT) of the *Tgfbr1* cKO mice, but not in those of the controls and tail (TL) DNA samples. (D to F) Relative *Tgfbr1* mRNA levels in the granulosa cells (D; n = 3), oviduct (E; n = 5), and uterus (F; n = 5) of control and *Tgfbr1* cKO mice. Data are presented as mean ± SEM. *P<0.05 compared with controls.

*Tgfbr1* null mice die embryonically [44], precluding functional characterization of TGFBR1 postnatally. To study TGFBR1–mediated signaling in female reproduction, we used a *Tgfbr1* ^flox^ allele and a *Tgfbr1* ^bgal^ allele, in which a β-galactosidase (β-gal) reporter was inserted into the *Tgfbr1* locus to create a null allele and to monitor spatiotemporal expression of *Tgfbr1*. To ensure maximal deletion of the *Tgfbr1* gene, the *Tgfbr1* ^bgal^ null allele was used in the breeding scheme to produce *Tgfbr1* mutant mice. Mice carrying these alleles were crossed with mice harboring the *Amhr2*-Cre allele [48], which recombines floxed alleles in granulosa cells [49] and Müllerian duct derived tissues (e.g., the smooth muscle layers of the uterus and oviduct but not the epithelial compartment) [50]–[52] to produce *Tgfbr1* cKO mice (*Tgfbr1* ^flox/bgal^; *Amhr2* ^cre/+^) (Figure 1A). Recombination of the *Tgfbr1* ^flox^ allele and reduction of *Tgfbr1* mRNA transcripts were confirmed in the ovary, oviduct, and uterus (Figure 1B–1F).

### *Tgfbr1* cKO Mice Are Sterile and Develop Prominent Oviductal Diverticula

Whereas control female mice (*Tgfbr1* ^flox/bgal^) lacking *Amhr2* ^cre/+^ demonstrated normal fertility and fecundity during a 6-month breeding period (8.5±0.2 pups/litter and 1.1±0.0 litter/month), the *Tgfbr1* cKO female mice were sterile (Table 1). Copulatory plugs were found in the *Tgfbr1* cKO females, indicating the infertility was not due to disrupted mating behavior. These results suggest TGFBR1 is required for female fertility.

**Table 1:** Fertility test of *Tgfbr1* cKO mice.

Genotype	n	Pups/litter	Litter/month
*Tgfbr1* ^flox/bgal^	10	8.5±0.2	1.1±0.0
*Tgfbr1* ^flox/bgal^; *Amhr2* ^cre/+^	7	0	0

The *Tgfbr1* cKO female mice were sterile during a 6-month breeding period compared with control female mice lacking *Amhr2* ^cre/+^, which showed normal fertility and fecundity. Data are presented as mean ± SEM.

Data are presented as mean ± SEM.

**Figure 2:**
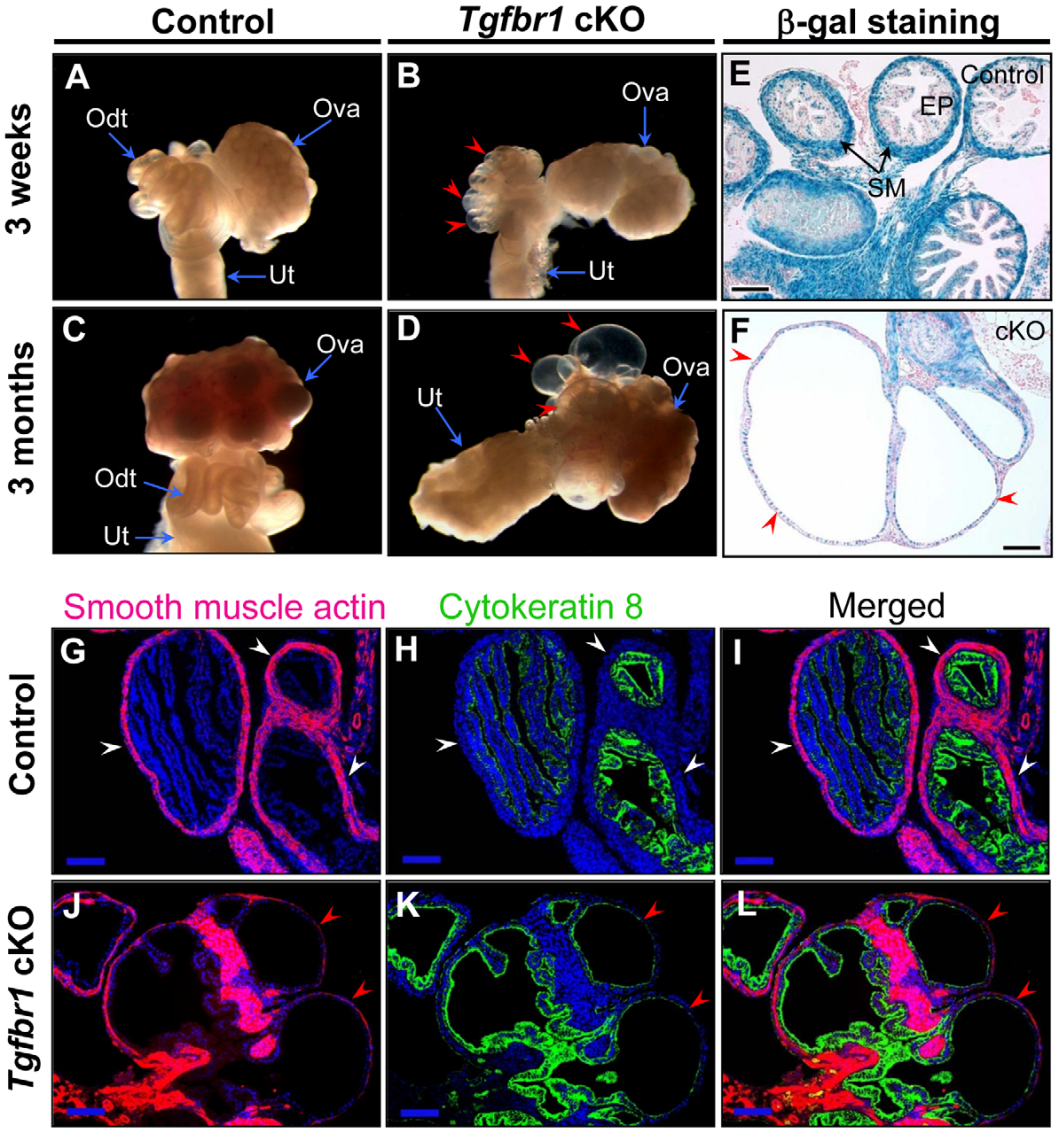
Oviductal diverticula development in *Tgfbr1* cKO mice. (A to F) Gross morphology (A to D) and β-gal staining (E and F) of oviducts or oviductal diverticula in control and *Tgfbr1* cKO mice. (G to L) Immunofluorescence of ACTA2 and KRT8 in the oviducts of 3-week-old control and *Tgfbr1* cKO mice. White arrowheads indicate the normal oviductal structure, while red arrowheads indicate oviductal diverticula. Odt, oviduct; Ova, ovary; Ut, uterus; SM, smooth muscle; EP, epithelium. Scale bars = 100 µm.

To examine the structural integrity of the reproductive tract and determine possible causes of sterility in the *Tgfbr1* cKO females, we performed morphological and histological analyses of *Tgfbr1* cKO and control mice. Strikingly, we found the development of bilateral oviductal diverticula (i.e., clear fluid-filled outpouchings that are present throughout the length of each oviduct) in 100% of the *Tgfbr1* cKO females examined (Figure 2A–2D). This phenotype highlights the importance of TGFBR1 in the oviduct where its expression was detected in both smooth muscle and epithelial compartments (Figure 2E). Deletion of *Tgfbr1* was expected only in the smooth muscle compartment due to the presence of *Amhr2*-Cre activity in the mesenchymal cells that give rise to the smooth muscle cells but not the epithelial cells. The oviductal diverticula enlarged with age and were characterized by a single layer of flattened epithelium and disrupted smooth muscle layers, as demonstrated by β-gal staining (Figure 2F) and immunofluorescence using antibodies against smooth muscle α-actin (ACTA2) and cytokeratin 8 (KRT8)(Figure 2G–2L) as well as calponin 1 (CNN1; Figure S1), a smooth muscle-specific protein implicated in contraction.

### *Tgfbr1* cKO Mice Demonstrate Minimal Ovarian Defects

**Figure 3:**
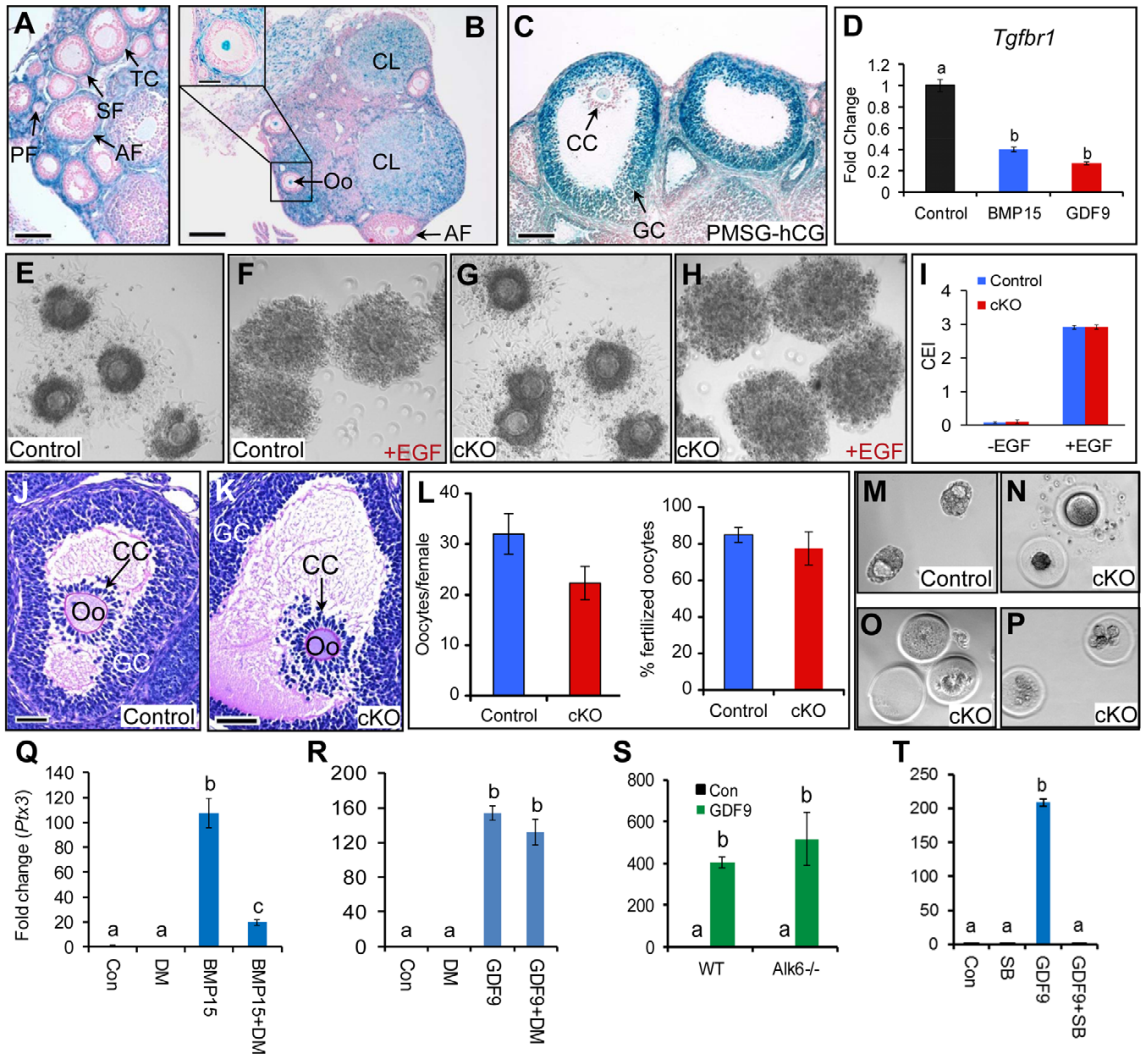
Cellular distribution and functional characterization of TGFBR1 in mouse ovary. (A to C) β-gal staining of ovaries from immature [(A; untreated) and (C; PMSG-hCG treated)] and adult (B) *Tgfbr1* ^+/bgal^ mice. (D) Suppression of *Tgfbr1* mRNA in mouse granulosa cells by recombinant BMP15 or GDF9 after 5 h treatment (n = 3). (E to H) Expansion of COCs from control and *Tgfbr1* cKO mice *in vitro* in the absence (E and G) or presence (F and H) of EGF (10 ng/ml). (I) Cumulus expansion index (CEI) of the *in vitro* cultured COCs from control (n = 4) and *Tgfbr1* cKO (n = 5) mice. (J and K) Preovulatory follicles from *Tgfbr1* cKO mice demonstrating cumulus expansion. PF, primary follicle; SF, secondary follicle; AF, antral follicle; Oo, oocyte; TC, thecal cell; GC, granulosa cell; CC, cumulus cell; CL, corpus luteum; COC, cumulus-oocyte complex. Scale bars = 50 µm (J and K); 100 µm (A and C); and 200 µm (B). (L) Ovulation and oocyte fertilization potential of *Tgfbr1* cKO mice (n = 8–10). (M to P) Blastocysts or degenerated oocytes/embryos recovered from control (M) and *Tgfbr1* cKO mice (N to P). (Q to T) Identification of receptor preference for GDF9 signaling in mouse granulosa cells using small molecule inhibitors and *Alk6* ^−/−^ granulosa cells. Dorsomorphin (DM; 4 µM) was preincubated with granulosa cells for 1 h before BMP15 (100 ng/ml) or GDF9 (15 ng/ml) was added. DM markedly reduced *Ptx3* induction by BMP15 (Q), while a similar effect was not observed on GDF9-induced *Ptx3* mRNA expression (R). Induction of *Ptx3* mRNA by GDF9 (100 ng/ml) was not attenuated in *Alk6^−/−^* granulosa cells (S). However, SB-505124 (SB; 1 µM) suppressed GDF9-induced *Ptx3* mRNA expression (T). Con, control. n = 3 for each group. Relative mRNA levels of *Tgfbr1* and *Ptx3* were normalized to *Gapdh*. Data are presented as mean ± SEM. Bars without a common letter are significantly different at *P*<0.05.

To define the causes of female sterility, we examined the ovaries of the *Tgfbr1* cKO mice. In contrast to the marked oviductal phenotype, the ovaries of *Tgfbr1* cKO mice were grossly normal and contained follicles at various follicular stages (Figure S2A and S2B). To address the cellular distribution of TGFBR1 in the mouse ovary, we performed β-gal staining and found that TGFBR1 was predominantly localized to the thecal layers of developing follicles (Figure 3A), corpora lutea (Figure 3B), oocytes (Figure 3B), and mural granulosa cells of preovulatory follicles induced by gonadotropins (Figure 3C). TGFBR1 expression signals in the granulosa cells of developing follicles and cumulus cells of preovulatory follicles were close to the background level (Figure 3A–3C). Furthermore, we found that GDF9 and its oocyte paralog BMP15 reduced the expression of *Tgfbr1* mRNA in mouse granulosa cells cultured *in vitro* (Figure 3D).

Because development of preovulatory follicles occurred in *Tgfbr1* cKO mice exposed to exogenous gonadotropins (Figure S2C and S2D), we examined cumulus expansion, a critical event in ovulation, in these follicles. We found that cumulus cells from the *Tgfbr1* cKO mice underwent normal expansion both *in vitro* and *in vivo* (Figure 3E–3K). We next conducted superovulation analysis to evaluate ovulatory potential and found that *Tgfbr1* cKO mice could ovulate although a trend of reduced ovulation rate was observed in these mice (Figure 3L). Similar to controls (Figure S3A), the ovaries of the superovulated *Tgfbr1* cKO mice contained corpora lutea (Figure S3B), which were capable of synthesizing 3-β-hydroxysteroid dehydrogenase (3β-HSD) (Figure S3D). As further evidence of the presence of functional corpora lutea in the *Tgfbr1* cKO mice, serum progesterone levels were comparable between the control and *Tgfbr1* cKO mice at 20 h after hCG injection (gonadotropin primed immature mice) or at 3.5 days post coitum (dpc; adult females) (Figure S3F). Moreover, oocytes could be located and recovered from the oviductal diverticula of the *Tgfbr1* cKO mice and were fertilizable (Figure 3L).

TGFBR1, also known as activin receptor-like kinase 5 (ALK5), had been proposed to mediate GDF9 signaling *in vitro* [35]. Based on the lack of a prominent ovarian phenotype in the *Tgfbr1* cKO mice and the minimal, if any, expression of TGFBR1 in the granulosa cells of preantral follicles, our results suggest that TGFBR1 is at least not the sole physiological type 1 receptor for GDF9 in mouse ovary. As an initial step toward exploring the potential type 1 receptor(s) for GDF9, we performed *in vitro* studies using *Alk6* null granulosa cells as well as small molecule inhibitors for ALK2/3/6 (Dorsomorphin; DM) and ALK4/5/7 (SB-505124; SB). While dorsomorphin potently suppressed BMP15-induced *Ptx3* expression as expected (Figure 3Q), a dramatic effect of this inhibitor on GDF9-induced *Ptx3* expression was not observed when GDF9 was applied at a concentration (15 ng/ml) that induced *Ptx3* mRNA expression with closer amplitude to that stimulated by 100 ng/ml of recombinant BMP15 (Figure 3R). Furthermore, GDF9 signaling remains intact in *Alk6* null granulosa cells as measured by the ability of GDF9 to induce the expression of cumulus expansion-related transcripts such as *Ptx3* (Figure 3S). In contrast, the ALK4/5/7 inhibitor, SB-505124, completely blocked the induction of *Ptx3* in mouse granulosa cells by GDF9 (Figure 3T). However, SB-505124 also reduced recombinant BMP15-induced *Ptx3* expression (data not shown), potentially due to its effect on basal gene expression in mouse granulosa cells. The above *in vitro* studies combined with our *in vivo* data support the hypothesis that GDF9 does not signal through type 1 BMP receptors (ALK2/3/6), but more likely signals through ALK4 and/or ALK7 in the mouse ovary.

### Deleterious Effects of Oviductal Diverticula on Female Fertility

Since ovulation and fertilization occurred in the *Tgfbr1* cKO and control females, we next assessed whether the formation of oviductal diverticula was detrimental to embryo development and/or transit of embryos to the uterus. After timed matings of adult *Tgfbr1* cKO females with proven fertile wild type (WT) males, we could recover blastocysts (7.75±0.63) at 3.5 dpc from the uteri of controls (Figure 3M), but not *Tgfbr1* cKO females (Table 2). Instead, degenerating oocytes/embryos and their zona pellucida remnants were recovered from the oviductal diverticula (Figure 3N–3P), indicating that embryo development and embryo transit to the uterus were severely compromised in the *Tgfbr1* cKO female mice. Since the oviduct is the site where sperm complete their maturation and undergo capacitation [53], sperm transport and/or capacitation could also be impeded in the adult *Tgfbr1* cKO mice due to the severe oviductal phenotype.

**Table 2:** Embryo recovery from 3.5 dpc uteri of control and *Tgfbr1* cKO mice.

Genotype	Dpc	n	No. of embryo
*Tgfbr1* ^flox/bgal^	3.5	4	7.75±0.63
*Tgfbr1* ^flox/bgal^; *Amhr2* ^cre/+^	3.5	5	0

After timed matings, blastocysts could be recovered at 3.5 dpc from the uteri of controls, but not *Tgfbr1* cKO females. Data are presented as mean ± SEM.

Data are presented as mean ± SEM.

### Loss of TGFBR1–Mediated Signaling Results in Defective Smooth Muscle Development in Mouse Uterus

**Figure 4:**
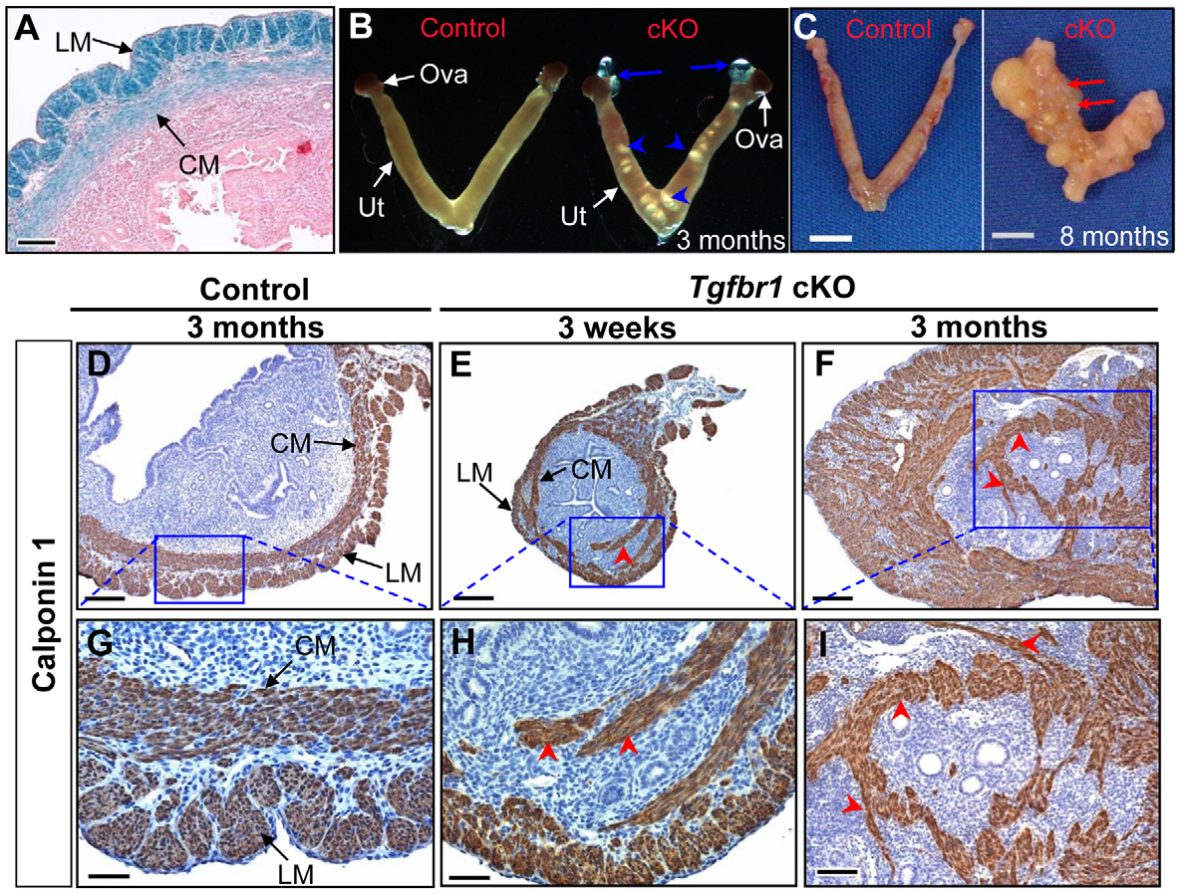
*Tgfbr1* cKO mice demonstrate disrupted uterine smooth muscle development. (A) Localization of TGFBR1 to myometrium by β-gal staining. (B and C) Gross uterine morphology of control and *Tgfbr1* cKO mice. Blue arrows and arrowheads indicate the respective oviductal diverticula and the smooth muscle defective areas, whereas red arrows point to uterine cysts. (D to I) Immunostaining of CNN1 in the uteri of control (D and G) and *Tgfbr1* cKO (E, F, H, and I) mice. LM, longitudinal muscle layer; CM, circular muscle layer; Ova, ovary; Ut, uterus. Red arrowheads point to disorganized uterine smooth muscle structures. Scale bars = 50 µm (G and H); 100 µm (A and I); 200 µm (D to F); and 5 mm (C).

Because TGFBR1 expression was also detected in smooth muscle cells of the uterus (Figure 4A) where *Amhr2*-Cre activity is present, we also examined the consequences of deletion of *Tgfbr1* in the uterus. Grossly, the uteri of the *Tgfbr1* cKO mice were comparable in size to those of controls up through 3 months (Figure S4A and S4B). However, the *Tgfbr1* cKO uteri contained multiple smooth muscle-defective areas, as evidenced by transillumination (Figure 4B). By 8 months of age, the uterine pathology in the *Tgfbr1* cKO mice culminated in uterine cyst formation and an almost unrecognizable mass of tissue (Figure 4C). The severely disrupted smooth muscle structure was evident by immunostaining of ACTA2 (Figure S4C–S4H) and CNN1 (Figure 4D–4I and Figure S5). In contrast to controls (Figure 4D and 4G), the myometrium of the *Tgfbr1* cKO mice was disorganized with poorly formed smooth muscle layers and intermingled with the endometrial components (Figure 4E, 4F, 4H, and 4I). Our data demonstrated that loss of TGFBR1–mediated signaling causes defective smooth muscle development in the oviduct and uterus.

To determine if the disorganization of uterine smooth muscle layers can affect stromal cell function, we performed an artificial decidualization study. Both the control and *Tgfbr1* cKO mice demonstrated responses to uterine scratches (Figure S6A and S6B). To quantitatively compare the decidual response between the control and *Tgfbr1* cKO groups, we calculated the weight ratio of stimulated (scratched) horn versus unstimulated horn. There was no significant difference between the two groups (Figure S6C; *P*>0.05), although disrupted uterine smooth muscle layers were visualized by immunostaining of CNN1 (Figure S6D–S6G). These results suggest that the compromised smooth muscle development in *Tgfbr1* cKO uterus does not prevent the uterine decidual response, although the decidualization occurs in an overall abnormal uterine environment where stromal cells were segregated by dispersed smooth muscle structure.

### Smooth Muscle Gene Expression in *Tgfbr1* cKO Oviducts

**Figure 5:**
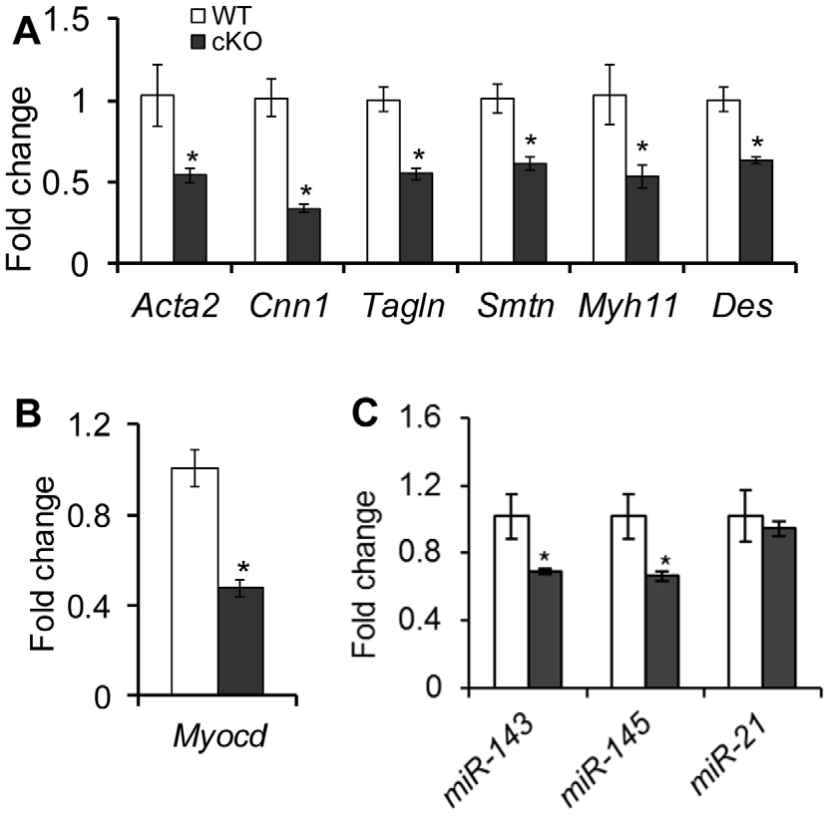
Dysregulation of smooth muscle genes and *miR-143* and *miR-145* in the oviducts of *Tgfbr1* cKO mice at 3–4 weeks of age. (A) Global reduction of smooth muscle gene transcripts in the oviducts of *Tgfbr1* cKO mice. (B) Down-regulation of *Myocd* mRNA in the *Tgfbr1* cKO oviducts. (C) Alterations of *miR-143* and *miR-145*, but not *miR-21*, in the oviducts of *Tgfbr1* cKO mice. Relative mRNA levels of smooth muscle genes (A) and *Myocd* (B) were normalized to *Gapdh*, while levels of *miR-143*, *miR-145*, and *miR-21* (C) were normalized against *snoRNA202*. n = 3–5 independent pools of oviducts. Data are presented as mean ± SEM. *P<0.05 versus controls.

To examine if loss of TGFBR1 affects the expression of smooth muscle genes, we compared the mRNA levels of select genes between control and *Tgfbr1* cKO oviducts from 3–4 week old mice. Accompanying the defective smooth muscle phenotype, transcript levels of all smooth muscle genes examined, including *Acta2*, *Cnn1*, transgelin (*Tagln*), smoothelin (*Smtn*), smooth-muscle myosin heavy chain (*Myh11*), and desmin (*Des*), were reduced in the oviducts of *Tgfbr1* cKO mice compared with controls (Figure 5A). Concomitantly, the mRNA level of myocardin (*Myocd*), a smooth muscle and cardiac muscle-specific transcriptional co-activator and a master modulator of smooth muscle gene expression [54], was also decreased in the *Tgfbr1* cKO oviducts (Figure 5B).

Interestingly, a similar oviductal phenotype was also observed in the *Amhr2*-Cre mediated conditional deletion of DICER1 [51], [52], [55], the RNase III involved in microRNA (miRNA) processing in cytoplasm. A recent study [56] demonstrated that TGFβ signaling can induce the maturation of a subset of miRNAs. An intriguing question was whether TGFβ signaling is linked to the miRNA pathway in the female reproductive tract. We found that two newly identified vascular smooth muscle associated miRNAs, *miR-143* and *miR-145* [57]–[60], were down-regulated in the oviducts of *Tgfbr1* cKO mice (Figure 5C). However, *miR-21*, a known target of TGFβ ligands [56], was not significantly affected in the *Tgfbr1* cKO oviducts (Figure 5C). Moreover, the downstream targets of *miR-143/145* (i.e., transcription factors *Elk1*, *Klf4*, and *Camk2d*) were not altered in the oviducts of *Tgfbr1* cKO mice (Figure S7).

Since the smooth muscle genes and *miR-143/145* were predominantly expressed in the smooth muscle compartments, loss of smooth muscle tissues in the oviducts during the formation of oviductal diverticula could potentially lead to reduced expression of smooth muscle genes or associated miRNAs in the *Tgfbr1* cKO oviducts. To address this possibility, we collected and analyzed postnatal day 7 oviducts prior to significant smooth muscle loss. However, dramatic reductions in the expression of smooth muscle genes and *miR-143* and *miR-145* were not detected at this stage (data not shown).

### *Tgfbr1* cKO Mice Develop a Uterine Phenotype Distinct from *Dicer1* cKO Mice

**Figure 6:**
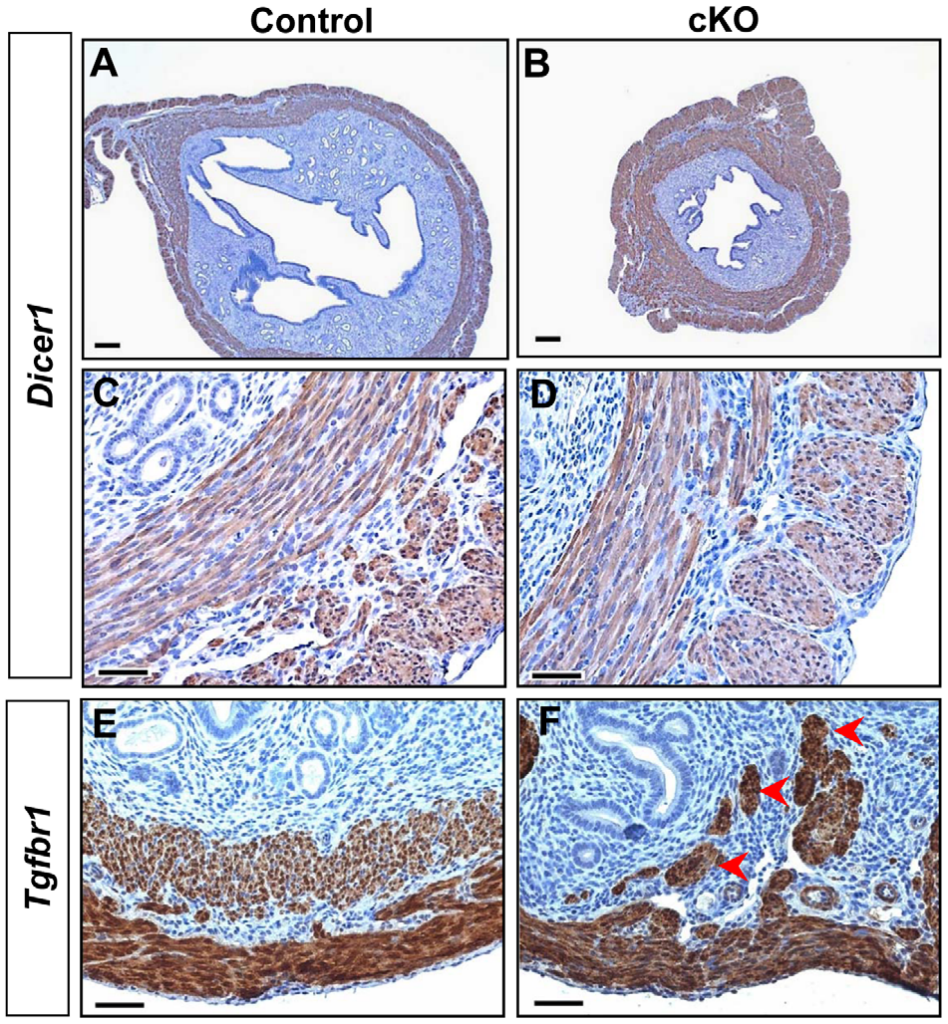
Distinct uterine phenotypes between *Dicer1* cKO and *Tgfbr1* cKO mice. (A–D) Adult *Dicer1* cKO mice (B and D) have smaller uteri but normal smooth muscle structure compared with controls (A and C) by immunostaining of uterine cross sections with CNN1. (E and F) Disruption of uterine smooth muscle layers in *Tgfbr1* cKO mice (F; arrowheads) versus controls (E) by immunostaining of uterine longitudinal sections with CNN1.

Despite the occurrence of oviductal diverticula in both *Tgfbr1* cKO and *Dicer1* cKO mice, we found that the uterine phenotype of *Tgfbr1* cKO mice is distinct from that of the *Dicer1* cKO mice. Consistent with our previous report [55], *Dicer1* cKO mice have smaller uteri than controls (Figure 6A and 6B), as is in contrast to *Tgfbr1* cKO mice. Unlike *Tgfbr1* cKO mice (Figure 6F), immunostaining showed that mice with a conditional deletion of *Dicer1* developed normal smooth muscle layers (Figure 6B and 6D). The divergence in the uterine phenotypes between *Tgfbr1* cKO and *Dicer1* cKO mice also suggests that the development of oviductal diverticula in these two mouse models may not be in a linear pathway. As further support of this concept, we found that mRNA levels for genes up-regulated in *Dicer1* cKO oviducts (*Wnt5a*, *Wnt7a*, *Hoxa9*, *Hoxa10*, etc.) [55] were not increased in *Tgfbr1* cKO mice (Figure S8).

### Molecular Alterations in the Oviduct of *Tgfbr1* cKO Mice

To further study the molecular basis of the striking oviductal diverticulum phenotype in *Tgfbr1* cKO mice, we performed real-time PCR analyses of oviducts from postnatal day 7 mice. We herein uncovered the dysregulation of candidate genes in the *Tgfbr1* cKO oviducts that are associated with cell differentiation (*Krt12* and *MyoR*) and migration (*Ace2*, *Vegfa*, and *Figf*  ).

**Figure 7:**
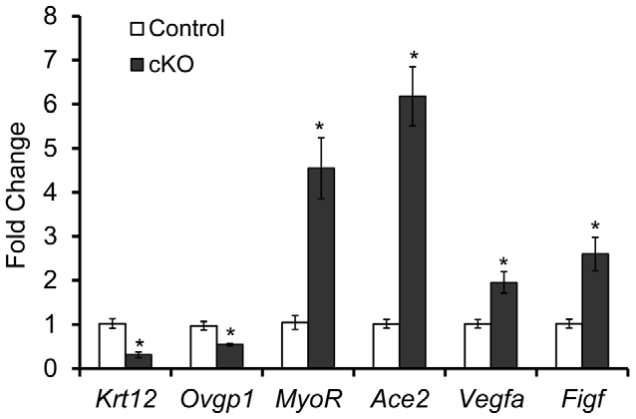
Dysregulation of genes in the oviducts from postnatal day 7 *Tgfbr1* cKO mice. Real-time PCR analyses using oviducts from postnatal day 7 mice demonstrated down-regulation of *Krt12* and *Ovgp1* and up-regulation of *MyoR*, *Ace2*, *Vegfa*, and *Figf* in *Tgfbr1* cKO mice (*n* = 5 independent pools of oviducts) versus controls (*n* = 5 independent pools of oviducts). Relative mRNA levels were normalized to *Gapdh*. Data are presented as mean ± SEM. *P<0.05 versus respective controls.

The keratins are intermediate filament proteins that are important structural components of epithelial cells [61], [62]. In the *Tgfbr1* cKO oviducts, expression of *Krt12*, a member of the keratins, was markedly reduced (*P*<0.05; Figure 7). Another epithelial gene, the oviductal glycoprotein 1 (*Ovgp1*), was also down-regulated (*P*<0.05; Figure 7). The altered expression of epithelial genes suggests the importance of TGFBR1–mediated signaling in the maintenance of the mesenchymal-epithelial interactions critical for oviductal development.

TGFβ signaling can regulate the differentiation of vascular smooth muscle cells [63]–[65]. We found that mRNA encoding the skeletal muscle differentiation associated basic helix-loop-helix (bHLH) transcription factor, *MyoR*/musculin, was increased more than 4-fold in *Tgfbr1* cKO oviducts versus controls (*P*<0.05; Figure 7).

Overexpression of angiotensin-converting enzyme 2 (ACE2), a member of the renin-angiotensin system, is associated with cell migration [66]. In the *Tgfbr1* cKO oviducts, the expression of *Ace2* mRNA was markedly increased in the *Tgfbr1* cKO oviducts (*P*<0.05; Figure 7). Even at 21 days of age, levels of *Ace2* mRNA were consistently higher in *Tgfbr1* cKO oviducts than controls (data not shown). Other migration related genes such as vascular endothelial growth factor A (*Vegfa*) and c-fos induced growth factor (*Figf*/*Vegfd*) were also up-regulated in the *Tgfbr1* cKO oviducts (*P*<0.05; Figure 7).

## Discussion

Despite the progress made on functional characterization of TGFβ family ligands in female reproduction, the *in vivo* roles of individual receptors in this pathway have remained elusive. Because conventional inactivation of *Tgfbr1* results in embryonic lethality [44], the functional understanding of this receptor in female reproductive tissues was hampered. In the current study, conditional deletion of *Tgfbr1* in the female reproductive tract using *Amhr2*-Cre expressed in granulosa cells and mesenchymal compartments of the oviduct and uterus [51], [55] led to female sterility. Histological analysis revealed that *Tgfbr1* cKO mice had minimal defects in their ovaries, which contain morphologically normal follicles at various developmental stages. To determine if the *Tgfbr1* cKO mice have normal ovarian function, we conducted superovulation and fertilization experiments. Our results showed that *Tgfbr1* cKO mice could ovulate, and the ovulated oocytes were fertilizable. These data suggest that TGFBR1 in mouse granulosa cells may not be essential for ovulation and oocyte fertilization.

TGFBR1 can mediate GDF9 signaling in granulosa cells *in vitro* [35]. Since GDF9 regulates folliculogenesis and cumulus cell expansion [12], [36], [42], [67], we were interested to know if cumulus cell function was impaired in the *Tgfbr1* cKO mice. We found that *Tgfbr1* cKO cumulus cells could expand *in vivo* and *in vitro*. Moreover, TGFBR1 was predominantly localized to thecal cells and corpora lutea, but not granulosa cells of developing follicles at preantral stage, the known sites for GDF9 action. After PMSG-hCG injection, the mural granulosa cells but not cumulus cells of preovulatory follicles highly expressed β-galactosidase from the *Tgfbr1* ^bgal^ knockin allele. Recombinant GDF9 or BMP15, another oocyte-derived factor implicated in follicular development [68]–[70], could markedly reduce *Tgfbr1* expression in mouse granulosa cells, which might explain the low intensity of TGFBR1 signals in cumulus cells adjacent to the oocyte. The data indicate that TGFBR1 might not be a physiological receptor for GDF9, or at least not the sole GDF9 type 1 receptor in mouse ovarian somatic cells. To further explore the potential GDF9 type 1 receptor(s) in mouse ovary, we utilized mouse granulosa cell culture and took advantage of *Alk6* null granulosa cells and small molecule inhibitors for ALK2/3/6 [71] and ALK4/5/7 [72]. Consistent with ALK6 as the BMP15 type 1 receptor [73], [74], the ability of recombinant BMP15 to induce cumulus expansion-related transcript expression is completely lost in mouse granulosa cells lacking ALK6 (data not shown). However, GDF9 signaling remains intact in *Alk6* null cells, excluding ALK6 as a GDF9 receptor. The small molecule inhibitor studies further helped to identify potential candidate receptors for GDF9 in mouse ovary (i.e., ALK4 and/or ALK7), although SB-505124 cannot precisely distinguish the type 1 receptor through which GDF9 signals. Despite these findings, future functional studies using conditional deletion of one or more type 1 receptors are needed to pinpoint the physiological receptor(s) for GDF9 in mouse ovary.

Because *Amhr2* is also expressed in mesenchyme-derived tissues in the oviduct and uterus [75], we examined whether the observed sterility is a phenotypic consequence of conditional knockout of *Tgfbr1* in the oviduct and/or uterus. We found that the *Tgfbr1* cKO mice develop a striking oviductal phenotype marked by the formation of bilateral diverticula. Histologically, a well-formed diverticulum comprises a single layer of smooth muscle cells and epithelium. The presence of degenerating oocytes/embryos in the oviductal diverticula but absence of blastocysts in the uteri (3.5 dpc) of the *Tgfbr1* cKO mice strongly indicate that development of oviductal diverticula is sufficient to cause female infertility in the *Tgfbr1* cKO mice, though it is plausible that disruption of the uterine smooth muscle development might sequentially confound the pregnancy outcome if pregnancy could occur in these mice. It is known that the myometrium plays an important role in key pregnancy-associated reproductive events, although current knowledge of myometrial causes of reproductive disorders is limited [76]. A successful labor is dependent on the synchronous myometrial contractions, which are regulated by a series of coordinated events at both hormonal and molecular levels during pregnancy [77], [78]. The disruption of uterine smooth muscle structure in the *Tgfbr1* cKO mice could potentially impede the contractility of the uterus or cause uterine rupture, with an adverse impact on pregnancy outcome. Emerging evidence suggests the involvement of the Wnt pathway in the maintenance of myometrium organization and integrity [79], [80]. Further investigations on the potential link between TGFBR1–mediated signaling and the Wnt pathway as well as the direct impact of the myometrial abnormalities resulting from loss of TGFβ/Wnt signaling components on reproductive potential may shed mechanistic light on reproductive disorders associated with smooth muscle pathology.

It is noteworthy that the oviductal phenotype of the TGFBR1-deficient mice resembles that of the conditional deletion of *Dicer1*, a key gene involved in miRNA and small interfering RNA (siRNA) biogenesis pathways [81]. MicroRNAs are non-coding small RNAs that regulate gene expression by inducing translational repression or mRNA degradation of target genes [81]. Recent studies in vascular smooth muscle cells suggest that TGFβ signaling can induce the maturation of a subset of miRNAs through the interactions between SMADs and the consensus RNA sequence of miRNAs within the DROSHA microprocessor complex [56], [82], [83]. Based on these findings and the similarity of the oviductal phenotype between *Tgfbr1* and our previously described *Dicer1* cKO mice [55], we proposed a potential link between the TGFβ signaling and miRNA pathways in the female reproductive tract. To test this hypothesis, we examined the expression of select genes/miRNAs in the oviducts of 3–4 week old *Tgfbr1* cKO and control mice. We found a global reduction of expression of smooth muscle genes, as well as two miRNAs, *miR-143* and *miR-145*, in the *Tgfbr1* cKO oviducts compared with controls. These two miRNAs are expressed in smooth muscle cells and have debatable roles in specifying smooth muscle phenotype [57]–[60], [84]. However, *miR-21*, which is regulated by TGFβ signaling in vascular smooth muscle cells [56], was not altered in the *Tgfbr1* cKO oviducts. The defective oviductal smooth muscle phenotype of the *Tgfbr1* cKO mice raised the possibility that the reductions of smooth muscle genes and smooth muscle associated miRNAs could be a consequence of reduced muscle components in the oviductal samples. To further address this question, we collected and analyzed oviductal samples from both control and *Tgfbr1* cKO mice at the age of 7 days prior to significant smooth muscle loss. We confirmed by quantitative PCR that *miR-143* was not significantly altered in the *Tgfbr1* cKO oviducts. Consistently, alteration of smooth muscle gene expression was not found in 7-day-old oviducts of *Tgfbr1* cKO mice. Therefore, the decreased expression of *miR-143/145* and smooth muscle genes in the 3–4 week old *Tgfbr1* cKO mice is likely caused by reduced smooth muscle components. Although *Dicer1* cKO mice develop oviductal diverticula [55], they have distinct uterine phenotypes (i.e., small uteri but histologically normal smooth muscle layers) and oviductal gene expression patterns compared to the *Tgfbr1* cKO mice. Moreover, the phenotype of *Tgfbr1* cKO mice is distinct from that of conditional deletion of *Smad2* and *Smad3* [18], suggesting the involvement of SMAD-independent pathway(s) downstream of TGFBR1. Collectively, the oviductal phenotype observed in *Tgfbr1* cKO mice is likely not a direct consequence of miRNA dysregulation.

Molecular analysis of the postnatal day 7 oviducts from *Tgfbr1* cKO mice demonstrated dysregulation of genes associated with cell differentiation and migration. Keratins have recently been highlighted as vital regulators of diverse cellular properties and functions (e.g., apico-basal polarization, motility, etc.), rather than simple epithelial markers [62]. KRT12 is a member of epithelial intermediate filament proteins which generally consist of two types of keratins (type 1 and type 2) as heterodimeric polymers [61], [62]. Dysregulation of epithelial genes in the oviducts of *Tgfbr1* cKO mice suggests that mesenchymal-epithelial interactions, which are potentially vital for smooth muscle development [85], could be affected when TGFBR1–mediated signaling is disrupted in the smooth muscle compartment although potentially functional TGFBR1 might still be present in the epithelial compartment due to the lack of *Amhr2*-Cre activity. As evidence of potentially altered smooth muscle cell differentiation, we found that MyoR/musculin was substantially up-regulated in the *Tgfbr1* cKO oviducts. Despite the fact that MyoR is also expressed in other cell types and can regulate their differentiation [86], the significance of MyoR up-regulation in *Tgfbr1* cKO oviducts awaits further investigation as current understanding of MyoR-regulated cell differentiation has been confined to the skeletal muscle lineage [87]. Beyond the aspects of cell differentiation, our data also point to the potential aberration of cell migration in the *Tgfbr1* cKO oviducts. It is well established that the renin-angiotensin system serves as a physiological system regulating blood pressure. Renin catalyzes the conversion of angiotensinogen to angiotensin I, which can be converted by ACE into angiotensin II. ACE2 is a newly described member of the renin-angiotensin system that can cleave angiotensin II into angiotensin 1–7, or angiotensin I into angiotensin 1–9 [66]. The renin-angiotensin system has been implicated in vascular smooth muscle cell proliferation and migration [88], [89], and ACE2 overexpression-induced alterations in cell migration have been documented [88]. In further support of aberrant cell migrations in the *Tgfbr1* cKO oviducts, we found increased expression of *Vegfa* and *Figf*/*Vegfd*. VEGFA and VEGFD are known regulators of smooth muscle cell migration [90], [91], and VEGF receptors are expressed in vascular smooth muscle cells [92]. Interestingly, *Vegf* is induced by TGFβ in mouse macrophages [93]. However, given the highly context-dependent nature of gene regulation by TGFβ signaling [94] as well as the diversity of ligands which signal via TGFBR1, it is not surprising that *Vegf* is up-regulated in mouse oviducts lacking TGFBR1. Moreover, it is not clear if the dysregulation of the aforementioned genes in the oviducts are direct or indirect effects of the loss of TGFBR1. Thus, our results indicate that profound molecular changes may occur in the smooth muscle and/or epithelial compartments of the oviduct in the absence of TGFBR1–mediated signaling. These alterations may developmentally affect the structural, migratory, and differentiating properties of the smooth muscle/epithelial cells, ultimately leading to the formation of the deleterious oviductal diverticula.

In summary, this study provides genetic evidence that TGFBR1–mediated signaling controls the integrity and function of the female reproductive tract. Disruption of TGFBR1–mediated signaling leads to catastrophic structural and functional consequences. Further in-depth understanding of the functional and regulatory significance of TGFBR1–mediated signaling in female reproductive physiology and pathology may help to discover novel therapeutic approaches for infertility treatment.

## Materials and Methods

### Ethics Statement

All mouse lines were manipulated according to the NIH Guide for the Care and Use of Laboratory Animals. All procedures have been approved by the Institutional Animal Care and Use Committee (IACUC) at Baylor College of Medicine. We took all necessary steps to minimize suffering of mice during the experiments.

### Animals and Cell Lines

All mouse lines were maintained on a mixed genetic background (C57BL/6/129S6/SvEv). The *Tgfbr1* ^flox^ allele was constructed by flanking the *Tgfbr1* exon 3 which encodes the transmembrane domain and the glycine/serine-rich (GS) domain with two *loxP* sites [44]. Mice harboring a *Tgfbr1* ^bgal^ allele (null allele) were generated and characterized by Deltagen and obtained from the Jackson laboratory. The function of the *Tgfbr1* gene was disrupted by insertion of a bacterial *LacZ* into the *Tgfbr1* gene (http://jaxmice.jax.org/strain/005847.html). The *Amhr2* ^cre/+^ mice were created by inserting a Cre-Neo cassette into the fifth exon of the *Amhr2* locus [48]. HEK-293 and HEK-293T cells were obtained from the Tissue Culture Core at Baylor College of Medicine. *Dicer1* cKO mice were generated as previously described [55].

### Production of Purified Recombinant BMP15 and GDF9

Recombinant human BMP15 was produced from HEK-293 stable cell lines as described previously [95]. Recombinant mouse GDF9 was constructed, produced, and purified using a similar strategy for engineering recombinant human BMP15 [95]. Briefly, we cloned the mouse GDF9 cDNA from 3-week old mouse ovaries. PCR-based mutations and introduction of restriction sites were performed using Phusion Hot Start High-Fidelity DNA polymerase (Finnzymes). Optimization of the cleavage and surrounding sequence (SHRSKRSLSGG) and introduction of a FLAG-tag (DYKDDDDK) were conducted by using overlap extension PCR. The genetically modified GDF9 sequence was cloned into pEFIRES-P, a bicistronic expression vector driven by human polypeptide chain elongation factor 1α promoter [96]. The GDF9 expression construct was then transfected into HEK-293T cells, and cell clones stably expressing recombinant GDF9 were selected in the presence of puromycin (Invitrogen) and used for the production of recombinant proteins. As a rigorous control for the purified recombinant GDF9, a “control buffer” was produced from the culture medium of non-transfected cells under the same purification approach as previously described [95].

### Generation of *Tgfbr1* cKO Mice

Using a *Cre-loxP* system [55], we first generated *Tgfbr1* ^+/bgal^; *Amhr2* ^cre/*+*^ mice. We subsequently crossed these mice with *Tgfbr1* ^flox/flox^ (homozygotes) to produce mice with the following genotypes: *Tgfbr1* ^flox/bgal^; *Amhr2* ^cre/+^ (cKO; experimental mice) and controls (*Tgfbr1* ^flox/bgal^ and *Tgfbr1* ^flox/+^). For the fertility tests, each female cKO or control mouse was caged with a WT male with known fertility at the age of 6 weeks for a 6-month period. The genotypes of the mice were analyzed by PCR using gene specific primers (Table 3).

**Table 3:** Primers for conventional PCR and SYBR green-based real-time PCR.

Name	Sequence (5′-3′)		Reference
*Tgfbr1* flox	Forward	ACTCACATGTTGGCTCTCACTGTC	[102]
	Reverse	AGTCATAGAGCATGTGTTAGAGTC	
*Tgfbr1* +/−	moIMR0003	GGGCCAGCTCATTCCTCCCACTCAT	*Tgfbr1* ^tm1Dgen^
	moIMR0843	CCTGTGGAGCTGGCAGCTGTCATTG	Jackson lab
	moIMR0844	ACTATCCGGGTCAGACAGCAAGCTC	
*Tgfbr1* Rec	Forward	ATTTCTTCTGCTATAATCCTGCAG	[102]
	Reverse	AGTCATAGAGCATGTGTTAGAGTC	
*Amhr2* Cre	Forward	CGCATTGTCTGAGTAGGTGT	[18]
	Reverse	GAAACGCAGCTCGGCCAGC	
*Hprt*	Forward	GGACCTCTCGAAGTGTTGGATAC	N/A
	Reverse	CTTGCGCTCATCTTAGGCTT	
*Tagln*	Forward	CGATGGAAACTACCGTGGAGA	[55]
	Reverse	TGAAGGCCAATGACGTGCT	
*Acta2*	Forward	CCACCGCAAATGCTTCTAAGT	[55]
	Reverse	GGCAGGAATGATTTGGAAAGG	
*Cnn1*	Forward	GGTGAAACCCCACGACATCTT	[55]
	Reverse	TTTGTCTTGGCCATGCTGG	
*Camk2d*	Forward	CAGCAGGCATGGTTTGGTT	N/A
	Reverse	CATAAGGATCTTTACGCAGGACTTC	
*Myh11*	Forward	CATCCTGACCCCACGTATCAA	[55]
	Reverse	ATCGGAAAAGGCGCTCATAGG	
*Des*	Forward	TACACCTGCGAGATTGATGCC	[55]
	Reverse	GCGCAATGTTGTCCTGATAGC	
*Smtn*	Forward	TCACTACCTTCAGCCATGCCT	[55]
	Reverse	GCCATTAGCTGCTTCCACTGT	
*Gapdh*	Forward	CAATGTGTCCGTCGTGGATCT	[55]
	Reverse	GCCTGCTTCACCACCTTCTT	
*Tgfbr1*	Forward	TGCCATAACCGCACTGTCA	N/A
	Reverse	AATGAAAGGGCGATCTAGTGATG	
*Myocd*	Forward	CCAACACCTTGCCCAGTTATC	N/A
	Reverse	GGAGCTTGTGCTGCCAAAG	
*Hoxa9*	Forward	CCTTCTCCGAAAACAATGCC	[55]
	Reverse	TCCTTCTCCAGTTCCAGCGT	
*Hoxa10*	Forward	TGGAGAAGGAGTTTCTATTCAACATG	N/A
	Reverse	TGGACGCTACGGCTGATCT	
*Elk1*	Forward	CATGACAGCCACGAATATGAAGA	N/A
	Reverse	TCTGACATATGCCATCATTTTGC	
*Klf4*	Forward	CTCACACAGGCGAGAAACCTT	N/A
	Reverse	GAGCGGGCGAATTTCCA	

Primer sequences are listed along with available references. All real-time PCR primers were designed using Primer Express software (Applied Biosystems).

### Histology and β-Gal Staining

For histological studies, mouse samples (ovaries, oviducts, and uteri) were fixed in 10% neutral buffered formalin (NBF) overnight. The samples were washed with 70% ethanol and embedded in paraffin by the Pathology Core Services Facility at Baylor College of Medicine. The samples were further processed for hematoxylin and eosin (H&E) or periodic acid Schiff (PAS)-hematoxylin staining using standard procedures.

For β-gal staining, mouse samples were fixed in fixation solution (2% paraformaldehyde, 0.2% glutaraldehyde, and 0.1 M phosphate pH 7.4) for 10–15 min. The samples were then rinsed 3 times for 30 min each in rinse buffer (0.01% sodium deoxycholate, 0.02% NP-40, 2 mM MgCl_2_, and 0.1 M phosphate pH 7.4). The β-gal staining was performed overnight at room temperature in staining buffer (0.01% sodium deoxycholate, 0.02% NP-40, 2 mM MgCl_2_, 5 mM potassium ferricyanide, 5 mM potassium ferrocyanide, 1 mg/ml X-gal, and 0.1 M phosphate pH 7.4). After staining, the samples were fixed in 10% NBF for at least 4 h, washed with 70% ethanol, and processed for sectioning. The sections were counterstained with fast red (Vector lab).

### Immunohistochemistry and Immunofluorescence

Paraffin-embedded sections (5 µm) were deparaffinized in xylene and rehydrated in graded alcohol. Antigen retrieval was performed by boiling the sections in 10 mM citrate buffer (pH 6.0) for 20 min. To quench endogenous peroxidase, the sections were treated with 0.3% (v/v) hydrogen peroxide, and then blocked with 3% goat serum for 30 min, followed by incubation with rabbit anti smooth muscle α-actin (Abcam; 1∶500) or rabbit anti-calponin 1 (Millipore; 1∶200) at 4°C overnight. After primary antibody incubation, the sections were washed and sequentially incubated with biotinylated anti-rabbit IgG and ABC reagent (Vector Labs). Immunoreactive signals were developed using a DAB substrate kit (Vector Labs). The sections were counterstained with hematoxylin.

Immunofluorescence was conducted using a similar protocol except that the hydrogen peroxide treatment was omitted and the secondary antibodies were Alexa fluor 488 (Invitrogen) and Alexa fluor 555 (Molecular Probes). The rat anti-cytokeratin-8 antibody was obtained from the Developmental Studies Hybridoma Bank (DSHB). The sections were mounted with Vectashield mounting medium containing DAPI.

### Superovulation, Embryo Culture, and Timed Mating

Superovulation experiments were performed to assess the ovulatory potential of the *Tgfbr1* cKO mice as described [18], [46]. In brief, the gonadotropin-primed female *Tgfbr1* cKO and control mice were mated with WT males. The cumulus-oocyte complexes (COCs) were recovered, exposed to M2 medium containing 1 mg/ml hyaluronidase, and counted. They were then cultured in M16 medium (Sigma). The number of embryos at the 2-cell stage was determined and recorded at the end of culture [55]. For timed matings, the control and *Tgfbr1* cKO females (6–8 week old) were mated with WT males, and copulatory plugs were checked the next morning. The oocytes or embryos were collected at 3.5 dpc from the oviducts or uteri.

### Hormone Analyses

Blood samples were collected by cardiac puncture from mice anesthetized with isoflurane. The serum was separated from the blood and stored at −20°C until assayed. Serum progesterone (P_4_) levels were measured by the Ligand Assay and Analysis Core at the Center for Research in Reproduction, University of Virginia. The detection limit was 0.1 ng/ml. Assay details can be found at http://www.healthsystem.virginia.edu/internet/crr/ligand.cfm.

### Cumulus Expansion Assays

Cumulus expansion analysis was conducted as previously described [18]. For *in vitro* cumulus expansion assay, the COCs were collected and cultured in the presence or absence of epidermal growth factor (EGF; 10 ng/ml). Cumulus expansion was examined after 16 h of culture and scored. The cumulus expansion index (CEI) was calculated based on the degree of COC expansion using a scale from 0 (no expansion) to 4 (complete expansion) [97]. For *in vivo* cumulus expansion analysis, immature mice (21–23 d) were treated with PMSG (46 h) and hCG (7 h). The ovaries were collected and processed for PAS staining, and the preovulatory follicles were examined microscopically for cumulus cell expansion.

### Primary Granulosa Cell Culture

Immature WT or *Alk6* null female mice [98], [99] were treated with 5 IU PMSG (i.p.). Large antral follicles were punctured to collect granulosa cells after 44–46 h [18], [95]. The cells were filtered through a 40 µm nylon mesh (Becton, Dickinson and Company), washed, and resuspended for the following experiments. (1) *Tgfbr1* mRNA regulation by oocyte-produced factors: The WT mouse granulosa cells were treated with control buffer [95], recombinant BMP15 (100 ng/ml), or recombinant GDF9 (100 ng/ml). The cells were collected in lysis buffer after 5 h treatment. (2) Gene induction assay (*Ptx3*): The WT and *Alk6* null granulosa cells were collected and treated with recombinant BMP15 (100 ng/ml) or GDF9 (100 ng/ml), and the cells were collected 5 h later. (3) Small molecule inhibitor assay: Mouse granulosa cells were preincubated for 1 h with dorsomorphin (Calbiochem; 4 µM) or SB-505124 (Sigma; 1 µM), and BMP15 or GDF9 was then added to the culture and further incubated for 5 h before the cells were collected. Total RNA was isolated from the cells harvested in the above experiments, and real-time PCR analyses were performed to determine the *Tgfbr1* or *Ptx3* mRNA expression.

### Reverse Transcription, Real-Time PCR, and microRNA Analysis

Total RNA from mouse granulosa cells, oviducts, or uteri was isolated using Qiagen RNeasy Micro or Mini Kit. Two hundred nanograms of total RNA were reverse transcribed using Superscript III reverse transcriptase (Invitrogen) [95]. Real-time PCR was performed using Taqman gene expression assay (Applied Biosystems) and Taqman PCR Master Mix or customized primers and SYBR green master mix [55]. Primer information is listed in Table 3 and Table 4.

**Table 4:** Taqman gene expression and miRNA assays.

miRNA	Taqman assay ID
*Tgfbr1*	Mm00436971_m1
*Ptx3*	Mm00477267_g1
*Wnt5a*	Mm00437347_m1
*Wnt7a*	Mm00437355_m1
*hsa-mir-143*	002249
*hsa-mir-145*	002278
*hsa-miR-21*	000397
*snoRNA202*	001232

Taqman assays were obtained from Applied Biosystems, and assay names and their corresponding IDs are listed.

For microRNA analysis, total RNA was isolated from mouse oviducts using mirVana miRNA isolation kit (Ambion). Levels of mature miRNA were measured using a two-step TaqMan MicroRNA Assay (Applied Biosystems). First, reverse transcription was performed using 10 ng of total RNA and stem-loop primers specific for *miR-143*, *miR-145*, and *miR-21*. Then, quantitative real-time PCR was conducted using specific Taqman probes for these miRNAs and Taqman universal PCR master mix (No AmpErase UNG).

Real-time PCR was carried out based on a protocol consisting of 40 cycles: 95°C for 10 min (hold), 95°C for 15 s (denature), and 60°C for 1 min (anneal/extend). All real-time PCR assays were performed in duplicate or triplicate for each sample. *Gapdh* was used as an internal control for the quantification of gene expression, while *snoRNA202* was used for normalization of miRNA levels. Relative mRNA abundance was calculated using ΔΔCT method [100].

### Uterine Decidualization

The uterine decidualization experiment was performed as described elsewhere [101]. Briefly, adult *Tgfbr1* cKO and control mice were subjected to ovariectomy followed by 3 days of estradiol treatment (100 ng/mouse) and 2 days of rest. The mice were subsequently treated with both estradiol (6.7 ng/mouse) and progesterone (1.0 mg/mouse) for 3 days. To artificially induce uterine decidualization, one uterine horn was traumatized/scratched using a needle on the antimesometrial side. The other uterine horn was not traumatized and used as a control. After that, the mice were continuously primed with estradiol and progesterone for 5 more days before sacrifice. Both uterine horns were weighed, and the tissues were collected and fixed in paraformaldehyde for histological and immunohistochemical assays.

### Statistical Analyses

A one-way analysis of variance (ANOVA) was applied to determine the difference of means among groups, and the difference between two means was further assessed by Tukey's HSD test. Comparison of means between two groups was conducted using *t*-test. Data are presented as mean ± standard error of the mean (SEM). Statistical significance was defined at *P*<0.05.

## Supporting Information

Figure S1 *Tgfbr1* cKO mice have defective smooth muscle formation in the oviduct. (A to C) Calponin 1 is expressed in the smooth muscle layers of the control oviduct (A and B). Negative control in which the primary antibody was substituted with rabbit IgG is shown in (C). (D to F) Defective smooth muscle formation in the oviducts of *Tgfbr1* cKO mice. Higher magnification view of the selected regions within the blue and red rectangles in (D) were depicted in (E) and (F), respectively. Arrowheads demonstrate smooth muscle defects. SM, smooth muscle; EP, epithelium. Scale bars = 50 µm (E and F); 100 µm (B and C); and 200 µm (A and D).
(TIF)

Figure S2 Follicular development in *Tgfbr1* cKO mice. (A and B) Ovaries of 3-week-old control and *Tgfbr1* cKO mice containing follicles at various developmental stages. (C and D) PMSG-induced follicular development in control (C) and *Tgfbr1* cKO (D) mice. Arrows indicate preovulatory follicles. Scale bars = 200 µm.
(TIF)

Figure S3 Corpora lutea formation in *Tgfbr1* cKO mice. (A and B) PAS staining of ovaries from PMSG-hCG treated control (A) and *Tgfbr1* cKO (B) mice. Corpora lutea formed in both control and *Tgfbr1* cKO mice after 46 h of PMSG and 20 h of hCG treatment. (C to E) Immunohistochemical staining of 3β-HSD in the corpora lutea of control (C) and *Tgfbr1* cKO (D) mice. A representative negative control of the *Tgfbr1* cKO mouse ovary was shown in (E). Scale bars = 200 µm. CL, corpus luteum. (F) Serum progesterone levels in gonadotropin-primed and natural pregnant mice (*n* = 3–4). Data are presented as mean ± SEM.
(TIF)

Figure S4 *Tgfbr1* cKO mice have defects in uterine smooth muscle formation. (A and B) Gross uterine morphology of *Tgfbr1* cKO mice at 3 weeks and 3 months of age. (C to H) Immunostaining of ACTA2 in the uteri of control (C and F) and *Tgfbr1* cKO (D, E, G, and H) mice. Note the disrupted smooth muscle layers in the *Tgfbr1* cKO mice, and the unusual appearance of the smooth muscle structure in the endometrium. Ut, uterus; LM, longitudinal muscle layer; CM, circular muscle layer. Red arrowheads point to the disorganized smooth muscle layers. Scale bars = 50 µm (F to H); 200 µm (C to E); and 5 mm (A and B).
(TIF)

Figure S5 Smooth muscle defects lead to severe disruption of uterine structures in *Tgfbr1* cKO mice. (A) Uterine abnormalities in an 8-month-old *Tgfbr1* cKO female compared to an age-matched control mouse. Blue arrows indicate the uteri. (B to F) Immunostaining of CNN1 in the uteri of 8-month-old control (B) and *Tgfbr1* cKO mice (C to F). CNN1 staining demonstrated multiple structural abnormalities in the uterine smooth muscle layers of the *Tgfbr1* cKO mice. Defective smooth muscle development leads to uterine cyst formation (arrow heads; C, D, and E), and complete loss of normal uterine structure in the severe cases (D and F). Scale bars = 200 µm (B to F).
(TIF)

Figure S6 The uteri of *Tgfbr1* cKO mice can undergo artificial decidualization. (A and B) Gross morphology of the uteri in the control (A) and *Tgfbr1* cKO (B) mice 5 days after the decidual stimulus. The left horns (control horns) of the uteri were unstimulated while the right ones (decidual horns) were traumatized. Note that the uteri of both control and *Tgfbr1* cKO mice can undergo artificially induced decidualization. (C) Weight ratio of decidual horn to control horn in control (n = 3) and *Tgfbr1* cKO mice (n = 4). Data are presented as mean ± SEM. (D–G) Immunostaining of control and decidual horns of *Tgfbr1* cKO mice using CCN1. Higher magnification views of (D) and (F) are depicted in (E) and (G), respectively. Note the disruption of smooth muscle structure in both unstimulated and decidual horns of the *Tgfbr1* cKO mice. Scale bars = 200 µm (E and G); 400 µm (D and F), and 10 mm (A).
(TIF)

Figure S7 Expression of target genes of *miR-143/145* in *Tgfbr1* cKO mice. Real-time PCR analyses using 3–4 week old oviducts demonstrated that *Elk1*, *Klf4*, and *Camk2d* are expressed at comparable levels between *Tgfbr1* cKO mice and controls. n = 3–4 independent pools of oviducts. Relative mRNA levels were normalized to *Gapdh*. Data are presented as mean ± SEM.
(TIF)

Figure S8 Messenger RNA levels of genes up-regulated in *Dicer1* cKO oviducts are not elevated in the oviducts of *Tgfbr1* cKO mice. *Wnt5a*, *Wnt7a*, *Hoxa9*, and *Hoxa10* are significantly up-regulated genes in the 3–4 week old oviducts of *Dicer1* cKO mice [55]. Real-time PCR analyses using age-matched *Tgfbr1* cKO and control oviducts did not demonstrate up-regulation of these genes in the *Tgfbr1* cKO mice. n = 3–4 independent pools of oviducts. Relative mRNA levels were normalized to *Gapdh*. Data are presented as mean ± SEM.
(TIF)
