# Supplementary figures and images for: Transforming Growth Factor β Receptor Type 1 Is Essential for Female Reproductive Tract Integrity and Function
Source: PLoS Genet. 2011 Oct 20;7(10):e1002320. doi: 10.1371/journal.pgen.1002320 (PMC3197682; doi:10.1371/journal.pgen.1002320)

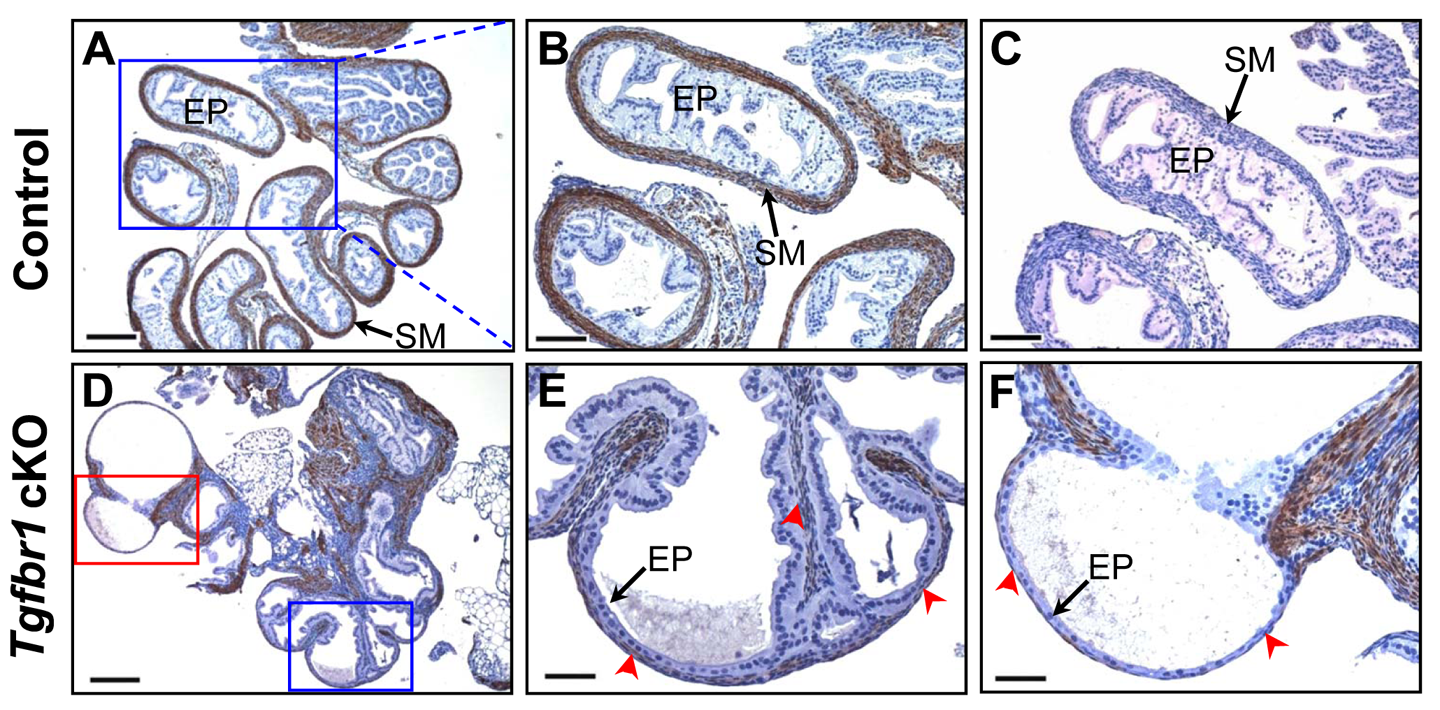

Supplement: Figure S1 — Tgfbr1 cKO mice have defective smooth muscle formation in the oviduct. (A to C) Calponin 1 is expressed in the smooth muscle layers of the control oviduct (A and B). Negative control in which the primary antibody was substituted with rabbit IgG is shown in (C). (D to F) Defective smooth muscle formation in the oviducts of Tgfbr1 cKO mice. Higher magnification view of the selected regions within the blue and red rectangles in (D) were depicted in (E) and (F), respectively. Arrowheads demonstrate smooth muscle defects. SM, smooth muscle; EP, epithelium. Scale bars = 50 µm (E and F); 100 µm (B and C); and 200 µm (A and D). (TIF) [file pgen.1002320.s001.tif]

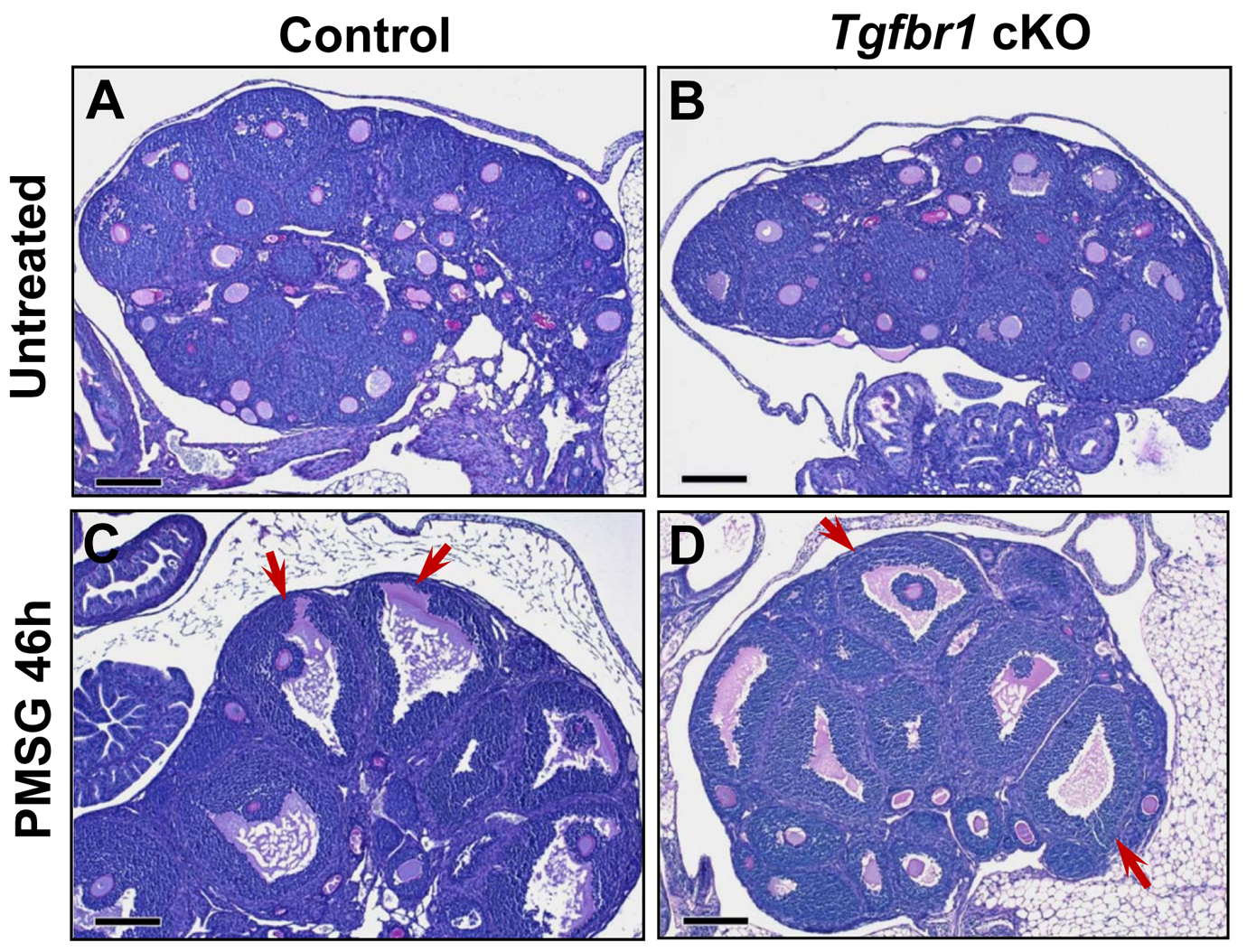

Supplement: Figure S2 — Follicular development in Tgfbr1 cKO mice. (A and B) Ovaries of 3-week-old control and Tgfbr1 cKO mice containing follicles at various developmental stages. (C and D) PMSG-induced follicular development in control (C) and Tgfbr1 cKO (D) mice. Arrows indicate preovulatory follicles. Scale bars = 200 µm. (TIF) [file pgen.1002320.s002.tif]

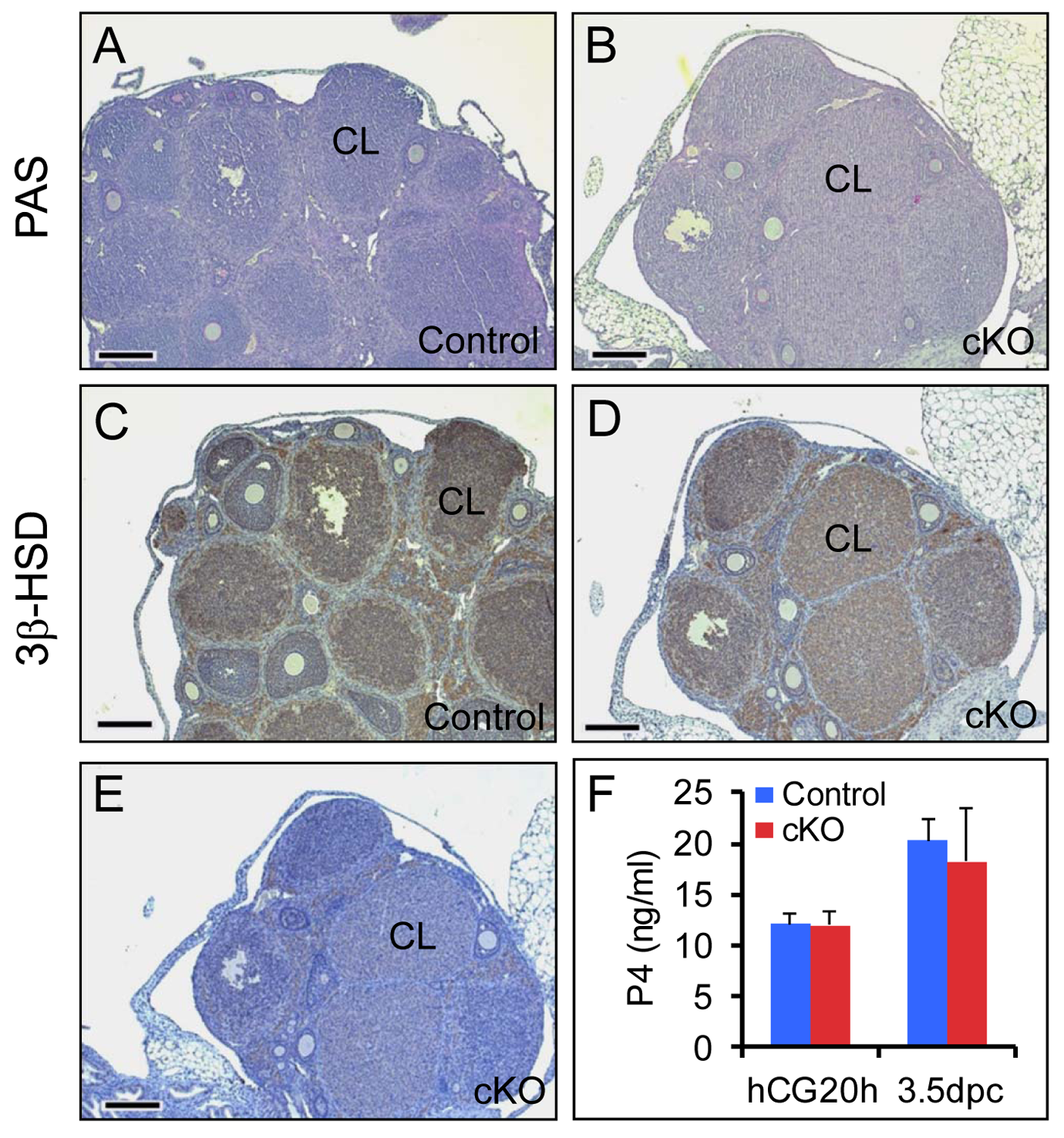

Supplement: Figure S3 — Corpora lutea formation in Tgfbr1 cKO mice. (A and B) PAS staining of ovaries from PMSG-hCG treated control (A) and Tgfbr1 cKO (B) mice. Corpora lutea formed in both control and Tgfbr1 cKO mice after 46 h of PMSG and 20 h of hCG treatment. (C to E) Immunohistochemical staining of 3β-HSD in the corpora lutea of control (C) and Tgfbr1 cKO (D) mice. A representative negative control of the Tgfbr1 cKO mouse ovary was shown in (E). Scale bars = 200 µm. CL, corpus luteum. (F) Serum progesterone levels in gonadotropin-primed and natural pregnant mice (n = 3–4). Data are presented as mean ± SEM. (TIF) [file pgen.1002320.s003.tif]

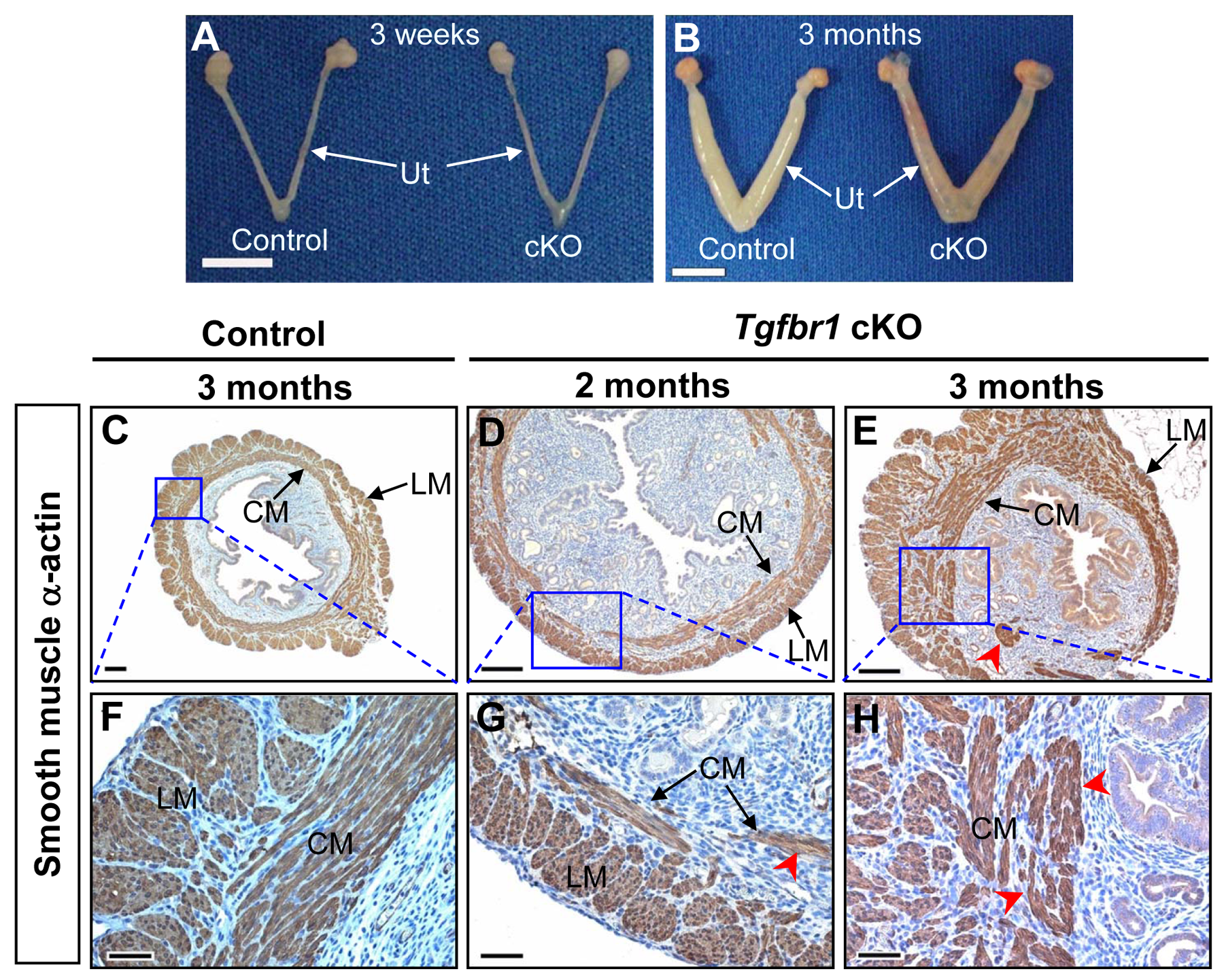

Supplement: Figure S4 — Tgfbr1 cKO mice have defects in uterine smooth muscle formation. (A and B) Gross uterine morphology of Tgfbr1 cKO mice at 3 weeks and 3 months of age. (C to H) Immunostaining of ACTA2 in the uteri of control (C and F) and Tgfbr1 cKO (D, E, G, and H) mice. Note the disrupted smooth muscle layers in the Tgfbr1 cKO mice, and the unusual appearance of the smooth muscle structure in the endometrium. Ut, uterus; LM, longitudinal muscle layer; CM, circular muscle layer. Red arrowheads point to the disorganized smooth muscle layers. Scale bars = 50 µm (F to H); 200 µm (C to E); and 5 mm (A and B). (TIF) [file pgen.1002320.s004.tif]

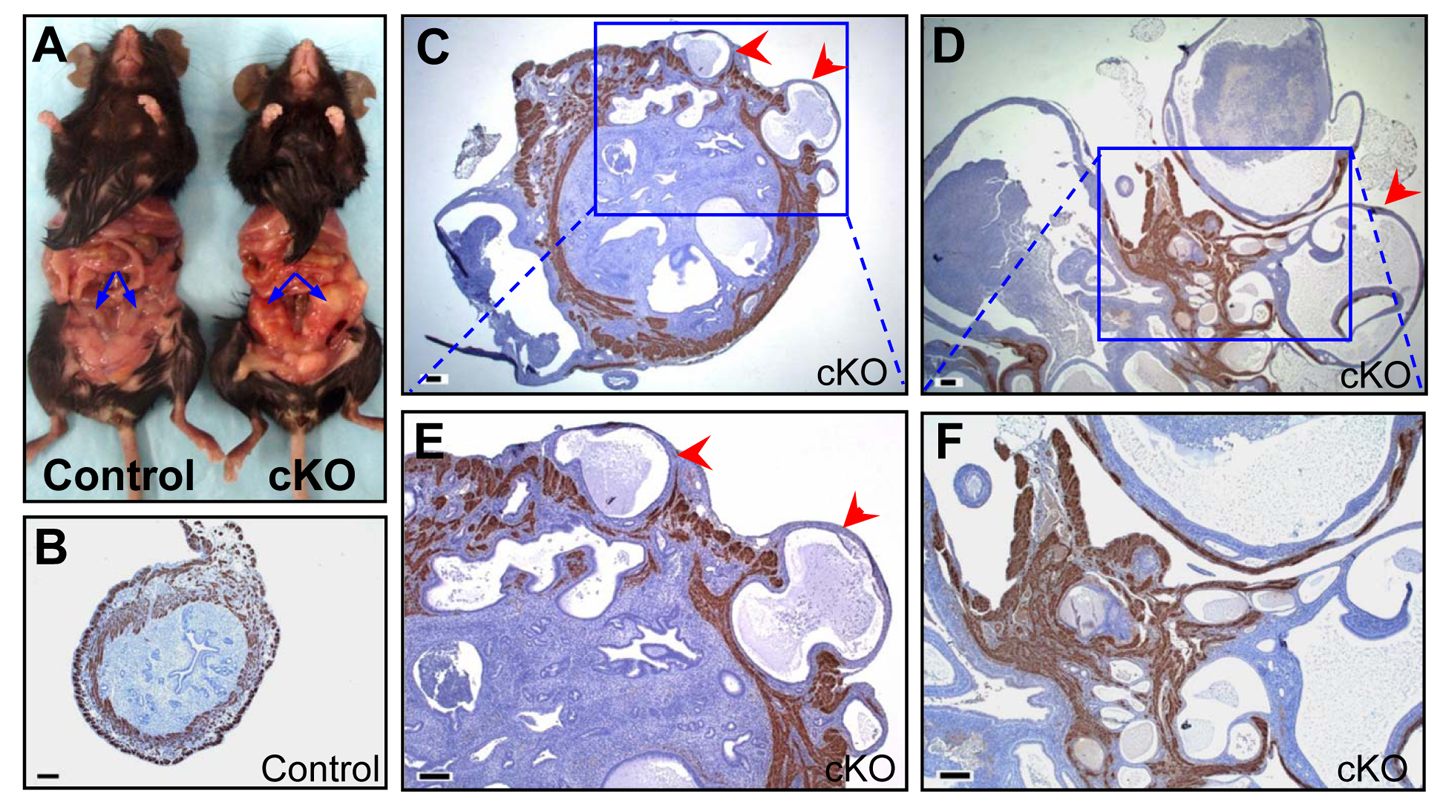

Supplement: Figure S5 — Smooth muscle defects lead to severe disruption of uterine structures in Tgfbr1 cKO mice. (A) Uterine abnormalities in an 8-month-old Tgfbr1 cKO female compared to an age-matched control mouse. Blue arrows indicate the uteri. (B to F) Immunostaining of CNN1 in the uteri of 8-month-old control (B) and Tgfbr1 cKO mice (C to F). CNN1 staining demonstrated multiple structural abnormalities in the uterine smooth muscle layers of the Tgfbr1 cKO mice. Defective smooth muscle development leads to uterine cyst formation (arrow heads; C, D, and E), and complete loss of normal uterine structure in the severe cases (D and F). Scale bars = 200 µm (B to F). (TIF) [file pgen.1002320.s005.tif]

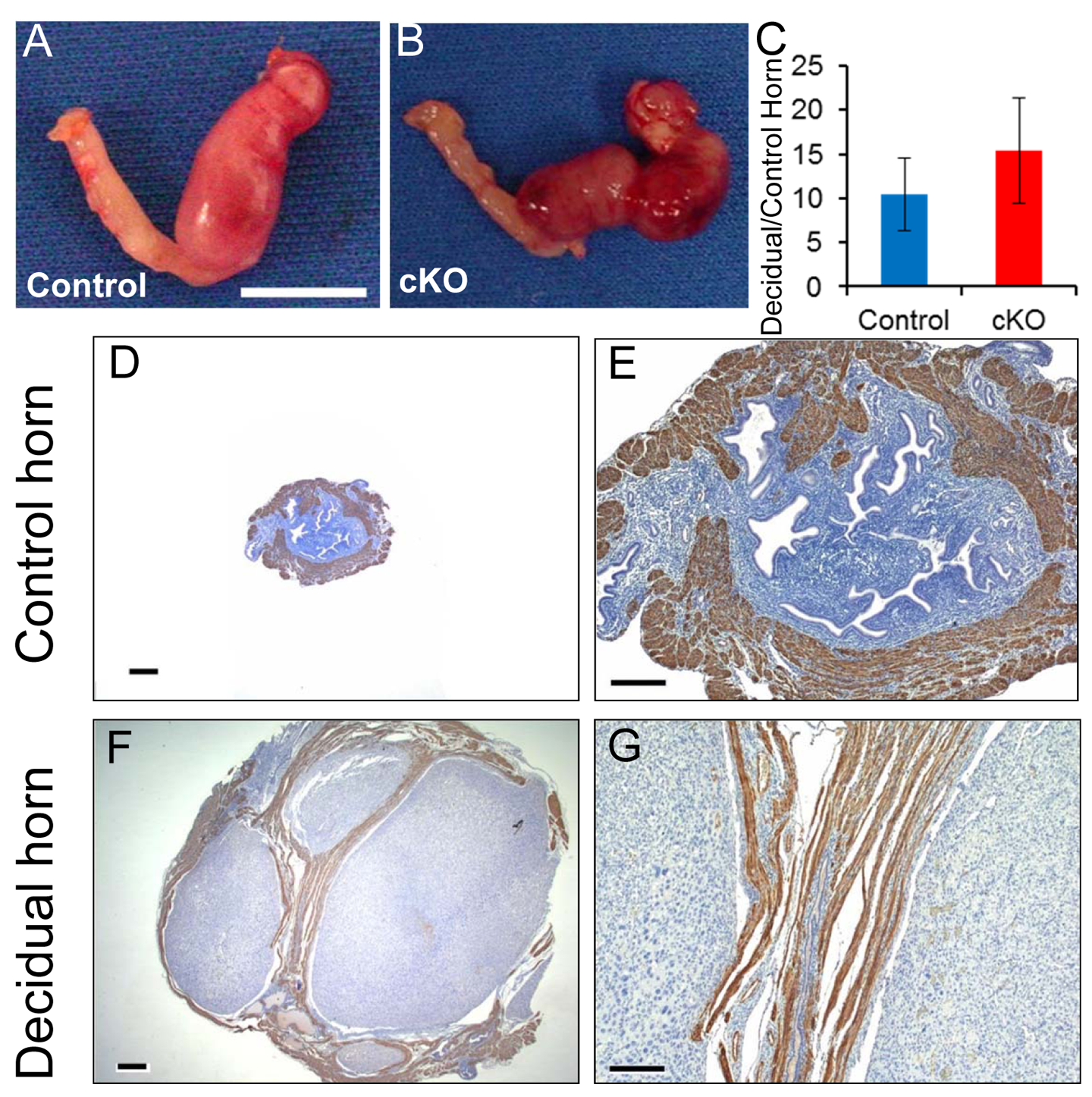

Supplement: Figure S6 — The uteri of Tgfbr1 cKO mice can undergo artificial decidualization. (A and B) Gross morphology of the uteri in the control (A) and Tgfbr1 cKO (B) mice 5 days after the decidual stimulus. The left horns (control horns) of the uteri were unstimulated while the right ones (decidual horns) were traumatized. Note that the uteri of both control and Tgfbr1 cKO mice can undergo artificially induced decidualization. (C) Weight ratio of decidual horn to control horn in control (n = 3) and Tgfbr1 cKO mice (n = 4). Data are presented as mean ± SEM. (D–G) Immunostaining of control and decidual horns of Tgfbr1 cKO mice using CCN1. Higher magnification views of (D) and (F) are depicted in (E) and (G), respectively. Note the disruption of smooth muscle structure in both unstimulated and decidual horns of the Tgfbr1 cKO mice. Scale bars = 200 µm (E and G); 400 µm (D and F), and 10 mm (A). (TIF) [file pgen.1002320.s006.tif]

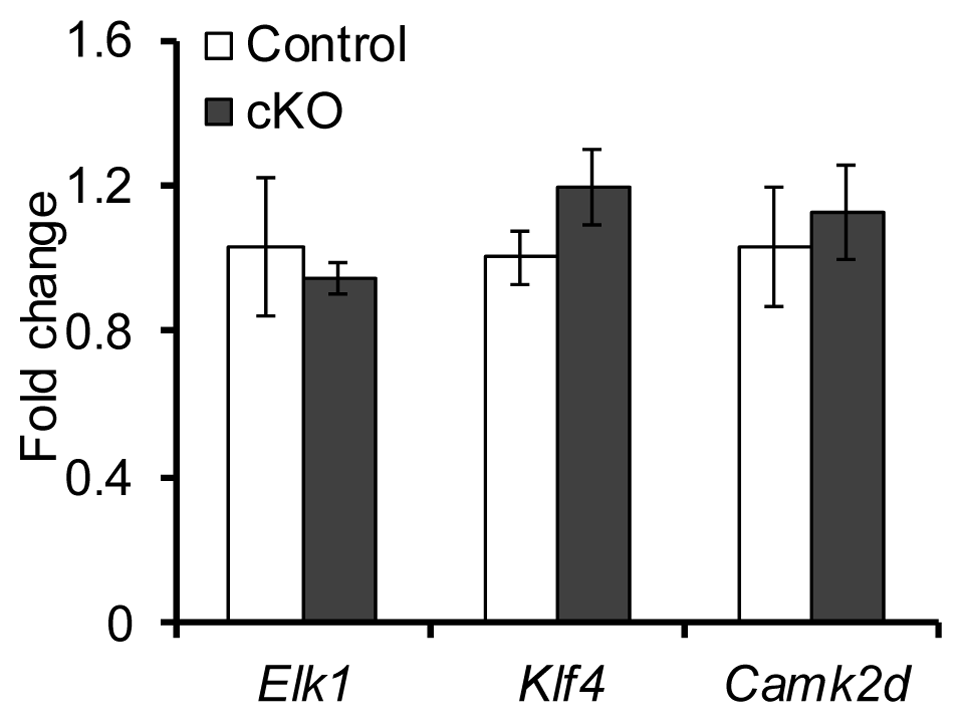

Supplement: Figure S7 — Expression of target genes of miR-143/145 in Tgfbr1 cKO mice. Real-time PCR analyses using 3–4 week old oviducts demonstrated that Elk1, Klf4, and Camk2d are expressed at comparable levels between Tgfbr1 cKO mice and controls. n = 3–4 independent pools of oviducts. Relative mRNA levels were normalized to Gapdh. Data are presented as mean ± SEM. (TIF) [file pgen.1002320.s007.tif]

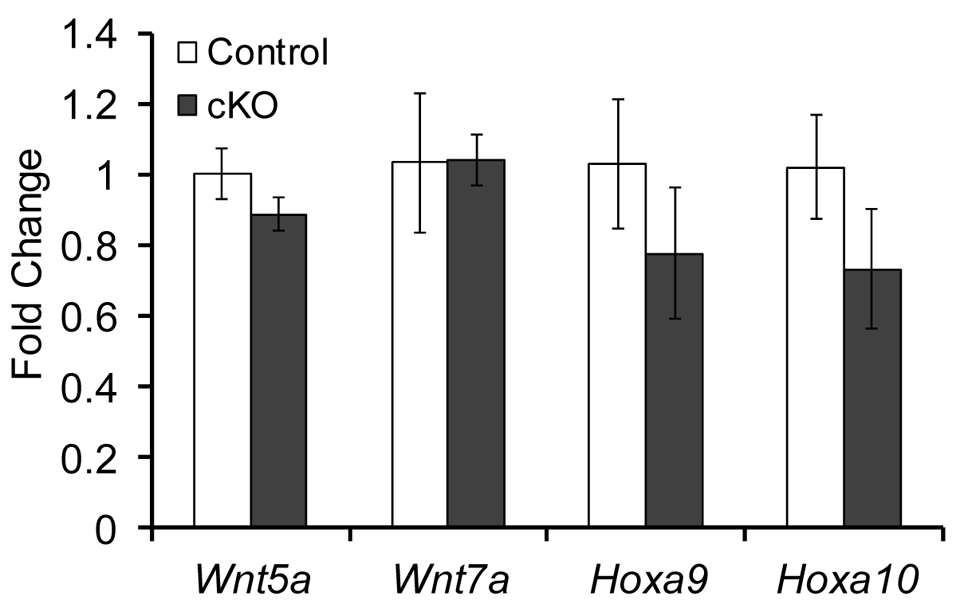

Supplement: Figure S8 — Messenger RNA levels of genes up-regulated in Dicer1 cKO oviducts are not elevated in the oviducts of Tgfbr1 cKO mice. Wnt5a, Wnt7a, Hoxa9, and Hoxa10 are significantly up-regulated genes in the 3–4 week old oviducts of Dicer1 cKO mice [55]. Real-time PCR analyses using age-matched Tgfbr1 cKO and control oviducts did not demonstrate up-regulation of these genes in the Tgfbr1 cKO mice. n = 3–4 independent pools of oviducts. Relative mRNA levels were normalized to Gapdh. Data are presented as mean ± SEM. (TIF) [file pgen.1002320.s008.tif]
